# Annexin A8 Identifies a Subpopulation of Transiently Quiescent c-Kit Positive Luminal Progenitor Cells of the Ductal Mammary Epithelium

**DOI:** 10.1371/journal.pone.0119718

**Published:** 2015-03-24

**Authors:** Juan Manuel Iglesias, Claire J. Cairney, Roderick K. Ferrier, Laura McDonald, Kelly Soady, Howard Kendrick, Marie-Anne Pringle, Reginald O. Morgan, Finian Martin, Matthew J. Smalley, Karen Blyth, Torsten Stein

**Affiliations:** 1 Institute of Cancer Sciences, College of Medical, Veterinary and Life Sciences, University of Glasgow, Glasgow, United Kingdom; 2 Synpromics Limited, Edinburgh, United Kingdom; 3 CRUK Beatson Institute, Glasgow, United Kingdom; 4 Medical Research Council Molecular Haematology Unit, Weatherall Institute of Molecular Medicine, University of Oxford, Oxford, United Kingdom; 5 European Cancer Stem Cell Research Institute, Cardiff School of Biosciences, Cardiff University, Cardiff, United Kingdom; 6 Institute of Molecular Cell and Systems Biology, University of Glasgow, Glasgow, United Kingdom; 7 Department of Biochemistry and Molecular Biology and the Institute of Biotechnology of Asturias (IUBA), University of Oviedo, Oviedo, Spain; 8 Conway Institute and School of Biomolecular and Biomedical Science, University College Dublin, Belfield, Dublin, Ireland; University of Tennessee Health Science Center, UNITED STATES

## Abstract

We have previously shown that Annexin A8 (ANXA8) is strongly associated with the basal-like subgroup of breast cancers, including BRCA1-associated breast cancers, and poor prognosis; while in the mouse mammary gland *AnxA8* mRNA is expressed in low-proliferative isolated pubertal mouse mammary ductal epithelium and after enforced involution, but not in isolated highly proliferative terminal end buds (TEB) or during pregnancy. To better understand ANXA8’s association with this breast cancer subgroup we established ANXA8’s cellular distribution in the mammary gland and ANXA8’s effect on cell proliferation. We show that ANXA8 expression in the mouse mammary gland was strong during pre-puberty before the expansion of the rudimentary ductal network and was limited to a distinct subpopulation of ductal luminal epithelial cells but was not detected in TEB or in alveoli during pregnancy. Similarly, during late involution its expression was found in the surviving ductal epithelium, but not in the apoptotic alveoli. Double-immunofluorescence (IF) showed that ANXA8 positive (+ve) cells were ER-alpha negative (−ve) and mostly quiescent, as defined by lack of Ki67 expression during puberty and mid-pregnancy, but not terminally differentiated with ∼15% of ANXA8 +ve cells re-entering the cell cycle at the start of pregnancy (day 4.5). RT-PCR on RNA from FACS-sorted cells and double-IF showed that ANXA8+ve cells were a subpopulation of c-kit +ve luminal progenitor cells, which have recently been identified as the cells of origin of basal-like breast cancers. Over expression of ANXA8 in the mammary epithelial cell line Kim-2 led to a G_0_/G_1_ arrest and suppressed Ki67 expression, indicating cell cycle exit. Our data therefore identify ANXA8 as a potential mediator of quiescence in the normal mouse mammary ductal epithelium, while its expression in basal-like breast cancers may be linked to ANXA8’s association with their specific cells of origin.

## Introduction

Annexins form a superfamily of calcium-dependent lipid-binding proteins broadly distributed throughout all eukaryotic phyla and even some bacteria and archea. These proteins feature unique homologous repeats that contain the calcium and lipid binding sites. However, their calcium-dependent lipid-binding ability is not a universal feature of annexins since some of them have partially or completely lost their type 2 calcium binding sites through evolutionary divergence [1]. This patterned structural diversity corresponds to a functional adaptation characteristic of individual subfamilies that ranges from membrane and cytoskeletal organisation to the regulation of membrane traffic and signalling. Annexins have also been shown to act as extracellular anti-inflammatory and anti-coagulant factors as cell surface proteins, and some have even been proposed to have nuclear roles [2–5]. They are further involved in phagocytosis as well as endo- and exocytosis (for reviews see [6–9]). Most initial studies have focused on their calcium-dependent membrane-binding properties but these may not be universal nor essential features for their action. Function-oriented studies have described annexins involved in cell growth and proliferation [10–12] and alterations of their expression have been associated with cancer subtypes and other diseases [13–16].

ANXA8 is one of the least characterised members of the annexin superfamily. ANXA8 was first described as an inhibitor of phospholipase A2 and as a blood coagulation factor (VAC-β) because of its structural similarity to VAC-α (ANXA5, lipocortin V) [17]. It was later found to be specifically over expressed in acute promyelocytic leukaemia (APL) where it was repressible by all-trans retinoic acid (ATRA) [18–21]. Deregulation of ANXA8 has since then been found in several other malignancies, including infiltrating adenocarcinomas of the pancreas [22], cholangiocarcinoma [23], malignant pleural mesothelioma [24], melanoma [25], squamous carcinoma of the uterine cervix [26], esophageal adenocarcinoma and Barrett’s metaplasia [27]. Perou et al. (2000) identified *AnxA8* by microarray analysis as part of an RNA signature for a subgroup of breast cancers with poor prognosis they called basal-like breast cancers because of their expression of basal cell associated cytokeratins (CK) 5 and 17 [28]. Our own work has previously established that ANXA8 protein is not detected in the majority of breast cancers but in a distinct subset of CK5 positive, oestrogen receptor (ER) α and progesterone receptor (PgR) negative breast cancers with poor prognosis and in a high percentage of BRCA1-associated cancers [29], confirming the RNA profiles by Perou et al. [28] and Sorlie et al. [30].

ANXA8 has been linked to the formation of endosomes and epidermal growth factor receptor (EGFR) turnover in Hela cells [31], and is required for efficient cell surface presentation of CD63 and P-selectin to allow leukocyte recruitment by activated endothelial cells [32]. Other studies identified ANXA8 as a target for p53-activated DNA damage response after treatment with adriamycin/doxorubicin of mouse embryonic fibroblasts [33] or when p53 was over expressed in Saos2 cells [34]. However its biological function in the mammary gland is still unknown.

We have previously shown that *Anxa8* mRNA was up-regulated during mouse mammary gland involution [29], a multi-step process in which the alveolar epithelium regresses by programmed cell death to a near pre-pregnant morphology [27, 32]. In the pubertal gland, *Anxa8* mRNA was found in enzymatically isolated epithelial ducts but not in terminal end buds [29]. In general, *Anxa8* mRNA abundance was highest during periods of widespread cell death or low proliferation. To get a better understanding of ANXA8’s role during mammary gland development we aimed to determine its cellular distribution at different developmental time points, to assess its association with different epithelial subpopulations, and to study the effect of ANXA8 expression *in vitro*.

Here we show for the first time that ANXA8 is expressed in a distinct quiescent subpopulation of ERα−ve cells of the ductal mammary epithelium during puberty and early pregnancy, but not in proliferating TEB or alveoli. During late involution, ANXA8 was only detected in the surviving epithelium, but not in the apoptotic cells. qRT-PCR using mRNA from FACS-sorted cells showed that AnxA8 was strongly associated with c-kit+ve/ERα−ve luminal progenitor cells (CD45^−^, CD24^+/high^, Sca1^−^, cd49f^−^, c-kit^+^), and triple-IF staining associated ANXA8 expression with a transiently quiescent subpopulation of the ductal luminal epithelium. Over expression in the mammary epithelial cell line KIM-2 altered the cell morphology and removed these cells from the cell cycle. Our data therefore strongly link ANXA8 to a subpopulation of c-kit+ve/ERα−ve ductal luminal epithelial progenitor cells and links ANXA8 function with cellular quiescence in the mammary epithelium. As this cell population was recently identified as the likely cells of origin for basal-like breast cancers, ANXA8’s expression in this cancer subgroup may therefore be a consequence of their cells of origin and thus a useful diagnostic marker.

## Materials and Methods

### Ethics statement

All animal work was conducted under project licence numbers PPL 60/3712 and PPL60/4181 in accordance with accepted standards of humane animal care and according to the UK Animals (Scientific Procedures) Act 1986 and the EU directive 2010 in dedicated facilities proactive in environmental enrichment. Ethical approval granted by University of Glasgow.

### Mammary gland preparation

The 4^th^ (inguinal) mammary glands were dissected and used for immunohistochemical staining or RNA extraction as described previously [35]. Balb/C mice were used unless stated otherwise.

### Cell Culture

KIM-2 cells were generated in the laboratory of C. Watson [36] and were maintained as previously described [36].

### *Anxa8* cloning into pRTS1

*Anxa8* cDNA was cloned into the pRTS1 episomal vector [37], a generous gift from Prof Bornkamm, before generation of KIM-2 cell lines that express *Anxa8* under the control of doxycycline. To generate the pRTS1:*Anxa8* construct, the mouse cDNA of *Anxa8* was cut from the IMAGE clone 5322310 using the restriction enzymes XhoI and EcoRV and ligated into the pUC19-SfiI vector using the XhoI and EcoRV restriction sites. The resulting construct was digested with Sfi1 and the cDNA fragment ligated into the Sfi1 sites of the pRTS1 vector. Positive clones were confirmed by sequencing. KIM-2 cells were transfected using FuGene 6 (Roche Applied Science, Burgess Hill, UK) according to manufacturer’s instructions with pRTS1:*AnxA8* and pRTS1 constructs and transfected cells were selected as pools using 250μg/ml of hygromycin (Calbiochem, Merck KG, Darmstadt, Germany) to establish Kim2A8 and Kim2RTS cell lines.

### Growth assay

Cells were plated in 24 well plates in triplicate and allowed to grow overnight before the addition of 100ng/ml doxycycline (Sigma-Aldrich, Gillingham, UK) at day 0. After washing the cells twice in PBS, protein lysates were prepared every 24 hours in Triton X-100 lysis buffer (20mM Tris pH 7.4, 250mM NaCl, 1% Triton X-100) plus Complete Mini Inhibitor mix (Roche Applied Science) and Protein Phosphatase Inhibitor Cocktail 2 (Sigma-Aldrich), and stored at −20°C. The protein concentration was measured using a BCA Protein Assay (Pierce, Thermo Fisher Scientific Inc, Rockford, USA).

### BrdU/EdU incorporation assay

Proliferation was measured using a Cell Proliferation ELISA, BrdU colourimetric kit (Roche Applied Science) in a 96 well format after 48 hours of doxycycline treatment (100ng/ml).

For *in vivo* EdU incorporation: C57Bl/6 mice were stud mated and allowed to lactate with standardized litter sizes of 5–6 for 7 days at which time forced involution was induced. The females were injected intra-peritoneally with 0.2ml of 5mg/ml EdU 2hr prior to sacrifice on day 4 after forced involution. For pre-pubertal samples EdU was injected in 3-week old females as above.

### Colony formation assay

Between 250 and 300 cells were seeded each in 10cm culture dishes in medium containing no doxycyline or 100ng/ml doxycycline. Cells were allowed to adhere and grow for 2 weeks, fixed with methanol, air dried and stained using Giemsa’s staining solution (BDH Laboratory Supplies, Merck Ltd., Lutterworth, UK). Pictures of the plates were taken using a digital camera and the number of colonies was quantified using ImageJ software.

### Cell cycle analysis by flow cytometry

Cells were grown in 6-well plates and treated with 100ng/ml doxycycline for 48 hours, trypsinized with Trypsin-EDTA, and collected together with any floating cells in the culture media. The cells were resuspended in 0.5ml PBS and fixed in 5ml 100% ice-cold methanol while vortexing. Cells were incubated for two hours at 4°C. After brief centrifugation, the methanol was removed and cells incubated in 400μl PI solution (50μg/ml propidium iodide, 50μg/ml RNAse A in PBS) for 30min before analysis on a BD FACSCanto II Flow Cytometer. Cyflogic software was used for analysis.

### Antibody production

The mouse *Anxa8* coding region was amplified by PCR using the primers A8-5’ (aatagaattcaatggcctggtggaaagcc) and A8-3’ (cgatctcgagtcagaggtcagtgcccac) and the IMAGE clone 5322310 as template. The PCR fragment was digested with EcoR1 and XhoI, cloned into pET302NT His vector (Invitrogen, Paisley, UK) digested with the same restriction sites and sequenced to verify that the 6xHis tag was in frame and the Anxa8 sequence was correct. This construct was used to produce ANXA8 protein in BL21 cells and the protein was purified using the TALON Metal Affinity Resin and Buffers (Clontech, Takara Bio Europe, Saint-Germain-en-Laye, France). After dialysis against PBS to remove imidazole, the purified protein was used to immunize two rabbits at EUROGENTEC (Fawley, Southampton, Hampshire, UK), using their standard protocol. The antiserum was affinity purified using recombinant His-tagged ANXA8 protein immobilized in a column generated using an AminoLink Plus Immobilisation Kit (Pierce). The antibodies were eluted from the column using 100mM glycine pH 2.5 and immediately neutralized by addition of 1/10 of the elution volume of 1M Tris. The specificity of the antibody was tested by western blot.

### Immunofluorescence and Immunohistochemistry

Cells were grown on eight-well chamber slides for the indicated times and fixed in 4% paraformaldehyde for 20 min at RT. After extensive washes with PBS, cells were incubated for 10 min in 50mM ammonium chloride followed by incubation for 10 min in 20mM glycine. Cells were incubated for 30–45 min in blocking solution (2.5% horse serum in PBS, 0.3% Triton X-100) to prevent nonspecific binding. The primary and secondary antibodies were diluted in blocking solution and antibody incubations were carried out at RT for 45–60 min. Washes were done with 0.1% Triton X-100 in PBS. Cells were finally washed in PBS before mounting the slides using Prolong Gold Antifade Reagent with DAPI (Molecular Probes, Invitrogen). Images were taken using an Olympus IX51 inverted microscope using a F-View camera and Cell^P 2.5 software (Olympus UK Ltd, Southend-on-Sea, Essex, UK). ImageJ software was used for image analysis.

For immunofluorescence (IF) on paraffin-embedded tissue, the sections were dewaxed in xylene and rehydrated through an alcohol gradient. Antigen retrieval was performed in 10 mM EDTA pH 8.0, sections were treated with Image-iT FX (Molecular Probes) for 30 min at room temperature and blocked with 2.5% horse serum in TBS-0.01% Tween 20. Tissue sections were stained in the same way as cells except that TBS-Tween 20 was used instead of PBS. Rat anti Ki67 clone TEC3 staining was developed by sequential incubation with the biotinylated secondary antibody from the rat ABC Staining System (Santa Cruz Biotechnology, Santa Cruz, CA, USA) at 1:100 and Streptavidin Dylight 488 (Pierce) at 1:200.

For immunohistochemistry on paraffin-embedded tissues, sections were treated in the same way as for IF, without the Image-iT FX step and the staining was developed using the ImmPRESS Peroxydase System (Vector Labs, Peterborough, UK).

Primary antibodies were used at the following concentrations; rabbit anti-Annexin A8 (Eurogentec) 1:100 for tissue and cells; mouse anti-ERα clone 6F11 (Leica Microsystems, Milton Keynes, UK) 1:70; rat anti-Ki67 clone TEC-3 (Dako) 1:50; goat anti-MCM3 (G19, Santa Cruz) 1:300; goat anti-mouse SCF R/c-kit (AF1356, R&D Systems Inc., Minneapolis, MN, USA,) 1:100 for IF. Donkey Alexa Fluor 488- and Alexa Fluor 594-labelled secondary antibodies (Molecular Probes) were used at a dilution 1:1000. For triple-staining, donkey anti-rabbit Alexa Fluor 488, donkey anti-goat-Cy5, and donkey anti-rat-Cy3 antibodies were used.

For EdU staining, a Click-iT EdU Alexa Fluor 595 Imaging Kit was used as per manufacturer’s instructions.

### Western blotting

Protein extracts were prepared using Triton X-100 lysis buffer plus Complete Protease Inhibitors (Roche Applied Science) and Protein Phosphatase Inhibitor Cocktail 2 (Sigma-Aldrich). The proteins were separated in NuPAGE 4–12% Bis-Tris gels and transferred to PROTRAN nitrocellulose transfer membranes (Whatman, Springfield Mill, Maidstone, Kent, UK). Membranes were blocked in 3% BSA diluted in TBS-Tween (20mM Tris pH7.6, 137mM NaCl, 0.1% Tween-20). The antibodies were diluted in blocking solution and incubated with the membrane in agitation for 2 hours at room temperature or overnight at 4°C and washed with TBS-Tween before developing with ECL Western Blotting Detection Reagents (GE Healthcare, UK Limited, Little Chalfont, Buckinghamshire, UK) in a FUJIFILM LAS-3000 Intelligent Dark Box (FUJIFILM UK Ltd, Bedford, UK). Antibodies were used at the following concentrations: rabbit anti-Annexin A8 (Eurogentec) 1:1000; rabbit anti-Ki67 (Abcam) 1:1000; goat anti-actin (C-11; Santa Cruz) 1:1000. Anti-goat secondary antibodies conjugated with horseradish peroxidase (DAKO, Glostrup Denmark) were used at 1:2000. Horseradish peroxidase-conjugated anti-rabbit IgG secondary antibody (GE Healthcare) was used at 1:5000.

### Cell Sorting

Primary mouse mammary cells were isolated by mechanical and enzymatic digestion as described [38]. Single cell suspensions at 10^6^ cells/ml were stained with anti-CD24-FITC (1.0μg/ml; BD Biosciences, Oxford, UK), anti-Sca-1-APC (1.0μg/ml; eBioscience, Hatfield, UK), anti-CD45-PE-Cy7 (1.0μg/ml; BD Biosciences), anti-CD49f-PE-Cy5 (5.0μl/ml; BD Biosciences) and anti-c-Kit-PE (1.0μg/ml; BD Biosciences). Cells were sorted on a FACSAria (Becton Dickinson, Oxford, UK) and mammary stem cells (MaSCs; CD45^−^, CD24^+/Low^, Sca-1^neg.^, CD49f^High^, c-Kit^−^), myoepithelial cells (CD45^−^ CD24^+/Low^, Sca-1^−^, CD49f^Low^, c-Kit^−^), luminal epithelial ERα−ve progenitors (CD45^−^, CD24^+/High^, Sca-1^−^, CD49f^−^, c-Kit^+^) and luminal epithelial ERα+ve differentiated cells (CD45^−^, CD24^+/High^, Sca-1^+^, CD49f^−^, c-Kit^−^) isolated using sort gates and controls as described [39].

Freshly sorted normal cells were resuspended in RLT buffer (Qiagen, Crawley, West Sussex, UK) and stored at −80°C until required for RNA extraction. qPCR reactions were performed as previously described [40] using TAQMAN Assays-on-Demand probe for *Anxa8* (Mm00507926_m1). *Actb* (β-actin) was used as an endogenous control and results calculated using the Δ-ΔC_t_ method. Data were expressed as the fold difference in gene expression between the mean of three independently isolated cell preparations compared to control samples with 95% confidence intervals.

### Quantitative RT-PCR on total mammary gland RNA

RNA from mammary glands was prepared using TRIZOL (Invitrogen) as described previously [35]. The RT reaction was carried out using 1μg of total RNA and Transcriptor reverse transcriptase (Roche Applied Science) following the guidelines of the supplier. For qPCR the following sets of primers and probes (Universal Probe Library from Roche Applied Science) were used to amplify *Anxa8* (ggaaaagcagcagacaggat, gagaactacccttcacgctgac, probe #31) and *Krt18* (agatgacaccaacatcacaagg, tccagaccttggacttcctc, probe #78) as internal control. The qPCR was performed using 1μl of cDNA as template, LC480 QPCR Master Mix (Roche Applied Science) and the appropriate set of primers in a 20μl reaction in a LightCycler 480 Instrument (Roche Applied Science).

## Results

### ANXA8 is expressed in a distinct subpopulation of luminal ductal epithelial cells

**Fig 1:**
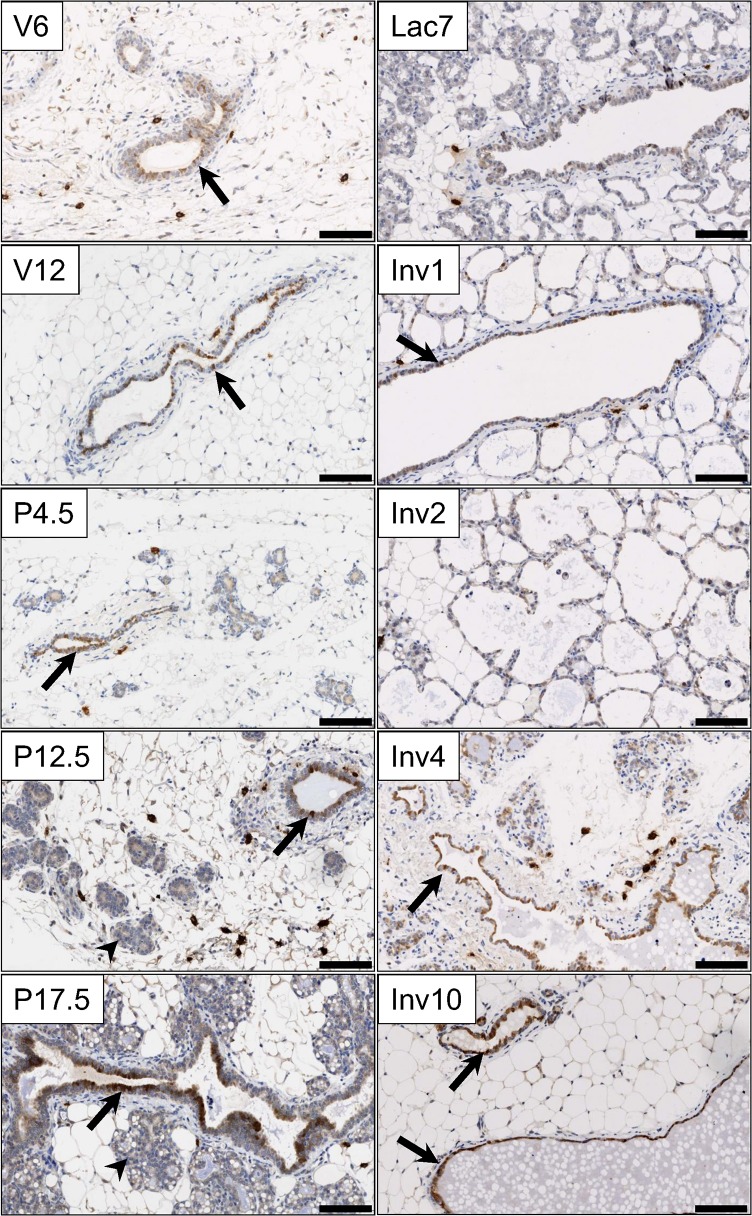
ANXA8 protein expression during mammary gland development. Sections from pubertal (6 weeks; V6), virgin (12 weeks, V12) pregnant (P, days 4.5, 12.5, 17.5), lactating (day 7) and involuting mice (days 1, 2, 4, and 10) were stained for ANXA8 protein. Staining was detected in a distinct set of ductal luminal epithelial cells (arrows), while alveoli (arrowheads) did not stain for ANXA8. ANXA8 was not expressed in the early involuting epithelium, but in the major ducts and widely in the surviving epithelium during late involution. The black bar represents 100μm.

To obtain an indication towards ANXA8’s role during mammary gland development it was necessary to assess its cellular distribution at different developmental time points. Since no antibodies were commercially available that recognised mouse ANXA8, a polyclonal antibody was raised and affinity-purified against full-length mouse ANXA8 protein, which showed specific reactivity in western blots with mouse ANXA8 but not with other annexins. Immunohistochemistry (IHC) detected ANXA8 protein specifically in a distinct subset of ductal luminal epithelial cells during puberty, adulthood, and pregnancy, and to a lesser extent in the major ducts during lactation (Fig. 1, S1 Fig.), while no ANXA8 was detectable in proliferating TEB or alveoli, or in differentiated alveolar epithelium. After enforced involution ANXA8 expression increased slowly and after four days was widely detected in major ducts and rarely in collapsed alveoli. After 10 days, ANXA8 was expressed in the majority of surviving ductal epithelial cells, which was consistent with the increased abundance of AnxA8 mRNA observed by qRT-PCR post-involution (S2 Fig.). In summary, ANXA8 expression was associated with a subpopulation of luminal ductal epithelial cells and with the surviving ductal epithelium during involution.

### ANXA8 expression is absent in highly proliferative mammary epithelium

**Fig 2:**
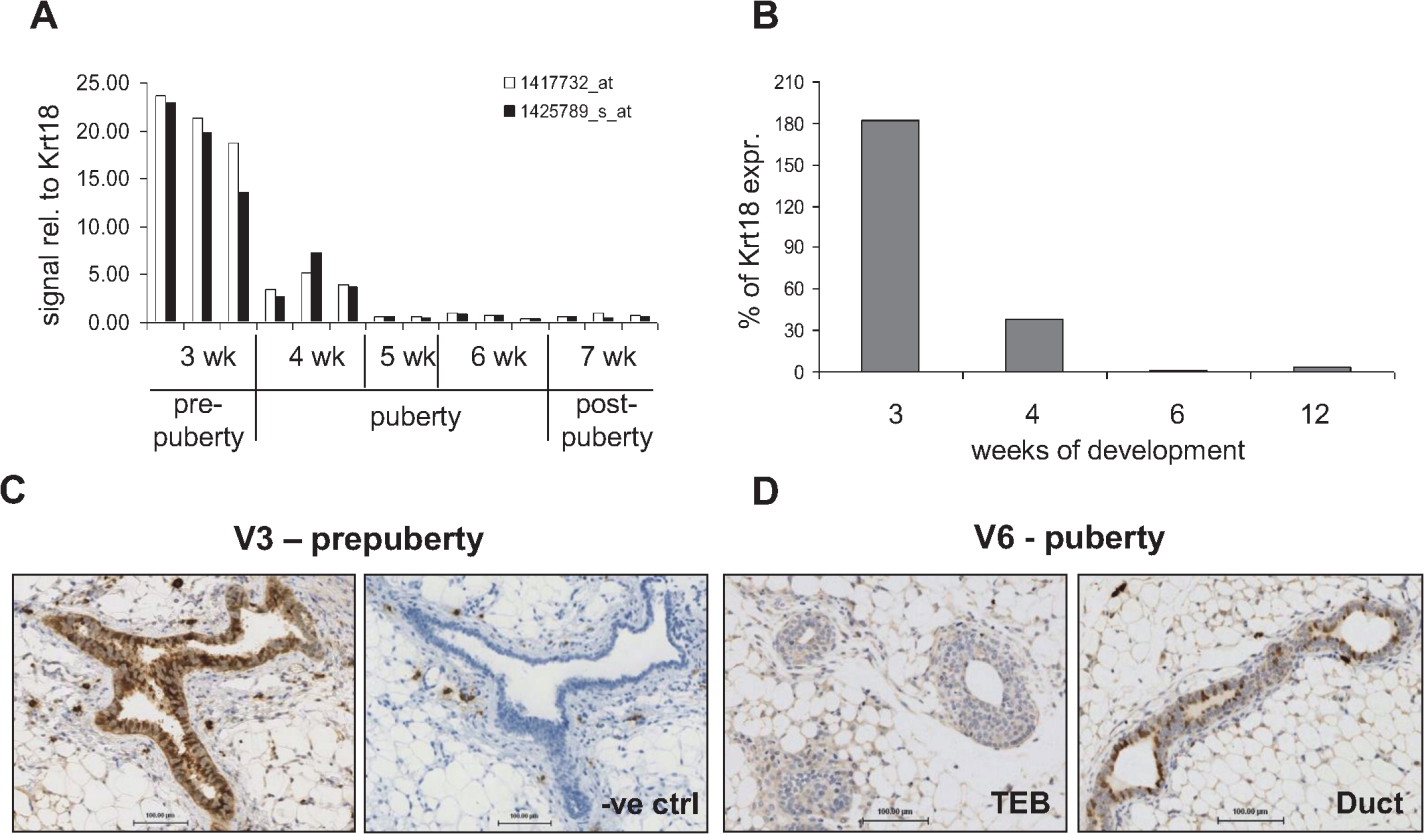
ANXA8 is expressed strongest during pre-puberty. (**A**) Microarray results from a previous microarray study [41], using RNA extracted from 3-, 4-, 5-, 6- and 7-week old CD1 mice, show a reduction in *AnxA8* mRNA at the onset of puberty. Signal intensities for two independent probes targeting *AnxA8* were normalised to cytokeratin 18 (Krt18) to eliminate changes due to differences in epithelial content. (**B**) qRT-PCR results for *AnxA8* normalised to Krt18 expression from RNA extracted from mammary glands of 3-, 4-, 6-, and 12-week-old mice. (**C-D**) Immunohistochemical analysis of ANXA8 expression using the E2R6.2 antibody on mammary glands from 3- (C) and 6-week old (**D**) mice showing staining for ANXA8 in the pre-pubertal rudiment and in ducts, but not TEB, of pubertal mice. Negative control (−ve ctrl): no primary antibody. Bars represent 100μm.

Analysis of data from a previous microarray study of pre-pubertal, pubertal and post-pubertal mouse mammary glands revealed that *Anxa8* mRNA abundance was highest during pre-puberty and strongly reduced at the onset of puberty (Fig. 2 (A)) [41] when the non-proliferative rudimentary ducts form proliferative TEB that grow out into the surrounding fat pad to establish the primary ductal mammary epithelial network. This reduction was confirmed by qRT-PCR using mRNA from 3-, 4-, 6-, and 12-week old mice, when normalised to the epithelial cell marker CK18 (Fig. 2(B)). IHC analysis showed again that ANXA8 was expressed in a distinct subpopulation of luminal epithelial cells of the pre-pubertal rudimentary epithelium (Fig. 2 (C)), as well as in individual cells of the ductal luminal epithelium in pubertal glands but never in TEB (Fig. 2 (D)). In contrast, Ki67 expression was widespread in TEB and in proliferating alveoli during pregnancy but rare in major ducts (S3 Fig.). ANXA8’s association with non-proliferative cells was further emphasised when 3-week old mice were injected with EdU for two hours (S4 Fig. (A)). Mammary glands from three independent mice showed no co-staining for ANXA8 and EdU. Highly proliferative regions, possibly by the onset of puberty, showed strong EdU staining, but no ANXA8 positivity, while ANXA8+ve ducts showed little EdU positivity with no overlap. Similar results were found in 4-day involuting glands, where strong ANXA8 staining but no EdU staining was observed in the surviving ductal epithelium (S4 Fig. (B)). Our results therefore associate ANXA8 with the low-proliferative rudimentary ductal epithelium, and show that its expression is switched off during pubertal outgrowth and proliferation.

### ANXA8 expressing epithelial cells are ERα−ve and transiently quiescent

**Fig 3:**
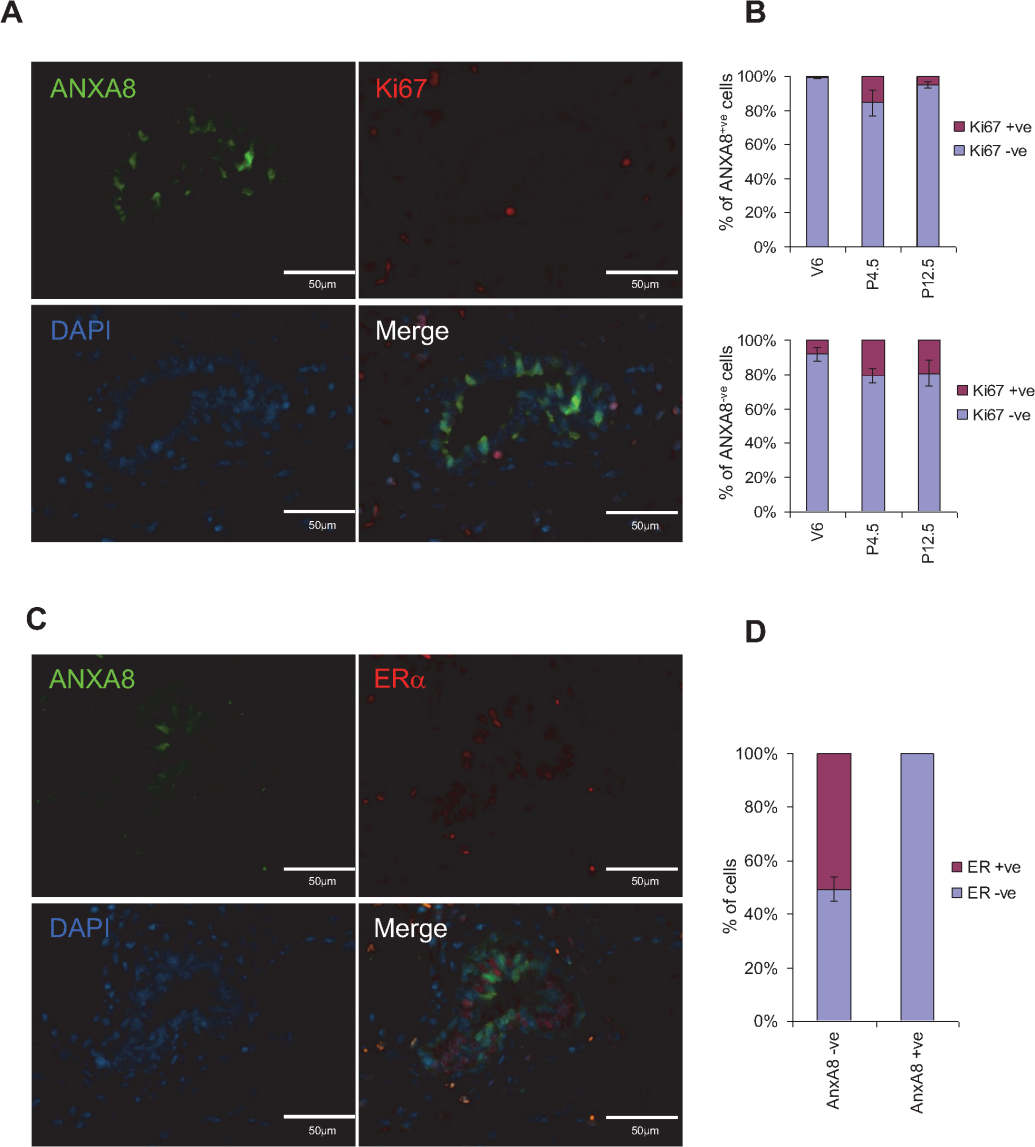
ANXA8 positive cells are ERα and Ki67 negative. Co-immunofluorescence staining for ANXA8 and Ki67 (**A**) or ERα (**C**) in 6-week old mice shows that those cells strongly positive for ANXA8 are negative for ERα and Ki67. Graphs represent the mean percentage of ANXA8 positive and negative cells that are positive or negative for Ki67 (B) or ERα (D) based on 1,000 cells per developmental time point (**B**) and at least 500 cells (**D**). V6: virgin 6 weeks; P4.5: pregnancy day 4.5; P12.5: pregnancy day 12.5. Error bars denote standard error of the mean.

Double-immunofluorescent (IF) labelling of pubertal mammary sections for ANXA8 and Ki67 established that over 99% of ANXA8+ve cells were quiescent with a lack of Ki67 expression (Fig. 3 (A, B)) and of the licensing factor MCM3 (S5 Fig.). However, the proportion of Ki67+ve/ANXA8+ve cells increased significantly during early pregnancy, reaching ∼15% of all ANXA8+ve cells (compared to ∼20% in ANXA8−ve ductal cells) but decreasing again to ∼5% during mid-pregnancy (day 12.5), while the proportion of cycling ANXA8−ve cells remained constantly high (∼19%; Fig. 3(B)). This demonstrated that ANXA8+ve cells were not all terminally differentiated, but were able to enter the cell cycle at the start of ductal budding, though ANXA8 was not detected in the newly formed epithelial structures.

Since it has previously been reported that ERα−ve cells of the mammary epithelium are the main proliferative compartment, the ERα-status of the ANXA8+ve cells was also established. Double-IF staining demonstrated that all ANXA8 expressing cells were in fact ERα−ve (Fig. 3 (C, D)), showing that ANXA8 was associated with a transiently quiescent ERα−ve subpopulation.

### AnxA8 mRNA is associated with c-kit+ve/ERα−ve luminal progenitor cells

**Fig 4:**
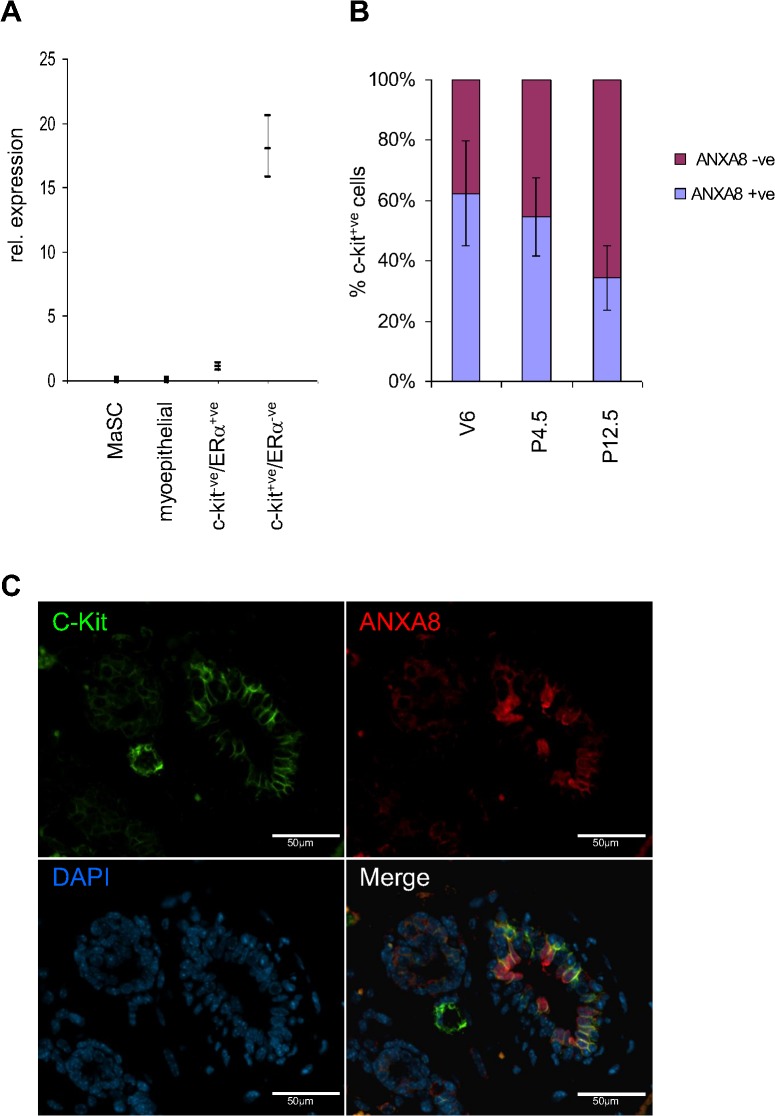
ANXA8 is expressed in ERα−ve/c-kit+ve luminal progenitor cells. (**A**) Primary mammary epithelial cells were sorted and RNA extracted as described previously [39]. qRT-PCR was used to measure *AnxA8* mRNA abundance in four different populations. *AnxA8* was not detected in either mammary stem cells (MaSC) or myoepithelial cells. Although very low levels were detected in differentiated ERα+ve luminal epithelial cells, ERα−ve luminal epithelial progenitor cells showed a 17-fold higher abundance. The graph shows the abundance relative to the levels of expression in differentiated cells and 95% confidence limits. (**B**) Bar graph showing the proportion of c-kit+ve/AnxA8+ve and c-kit+ve/AnxA8−ve cells during puberty (V6), early (P4.5) and late (P12.5) pregnancy. 1,000 cells were assessed per developmental time point. Error Bars denote standard error of the mean. (**C**) Co-immunofluorescence staining for ANXA8 and c-kit in mouse mammary gland from an early-pregnant (day 4.5) mouse.

ANXA8 is strongly expressed in BRCA1-associated breast cancers and these cancers have recently been shown to originate from ERα−ve luminal progenitor cells [42]. Since ANXA8 showed strong association with ERα−ve cells in the mammary gland it was hypothesised that ANXA8 was associated with the ERα−ve progenitor cell population. *AnxA8* mRNA expression was therefore measured by qRT-PCR in RNA from mammary epithelial cells that had been sorted according to their expression of the cell surface proteins CD24, CD49f, Sca1, and c-kit: a) mammary stem cells (MaSC; CD24^+/low^, Sca1^−^, CD49f^+/high^, c-kit^−^), b) myoepithelial cells (CD24^+/low^, Sca1^−^, CD49f^+/low^, c-kit^−^), c) mature luminal ERα+ve cells (CD24^+/high^, Sca1^+^, CD49f^−^, c-kit^−^), and ERα−ve luminal progenitor cells (CD24^+/high^, Sca1^−^, CD49f^−^, c-kit^+^) [39]. While no *AnxA8* mRNA was detectable in MaSC or myoepithelial cells, the luminal ERα−ve progenitor cell population had a 17-fold increased abundance compared to the differentiated luminal ERα+ve population (Fig. 4 (A)). The strong association of ANXA8 and c-kit expression was further emphasised by IF (Fig. 4 (B, C)). ANXA8 was co-expressed with c-kit in the luminal epithelium of mammary ducts, and localised to the cytoplasm as well as the apical and, similar to c-kit, to the lateral membranes (Fig. 4 (C)). However, co-expression of c-kit and ANXA8 varied within and between sections. While all ANXA8+ve cells were c-kit+ve independent of developmental stage, ANXA8 positivity of c-kit+ve cells ranged from as little as 0% to 100%. During puberty, strong c-kit staining was detected in the inner body cells of the TEB while ANXA8 could only be found in the ductal epithelium (S6 Fig.). During pregnancy ANXA8 and c-kit expression were both restricted to the ductal epithelium, though limited c-kit staining was found in the newly formed ductal outgrowth. The percentage of ANXA8+ve/c-kit+ve positive cells was reduced during pregnancy from just ∼62% to ∼34% (Fig. 4 (B)), possibly reflecting the overall reduction in ANXA8+ve cells as c-kit was still expressed in the majority of ductal luminal epithelial cells.

**Fig 5:**
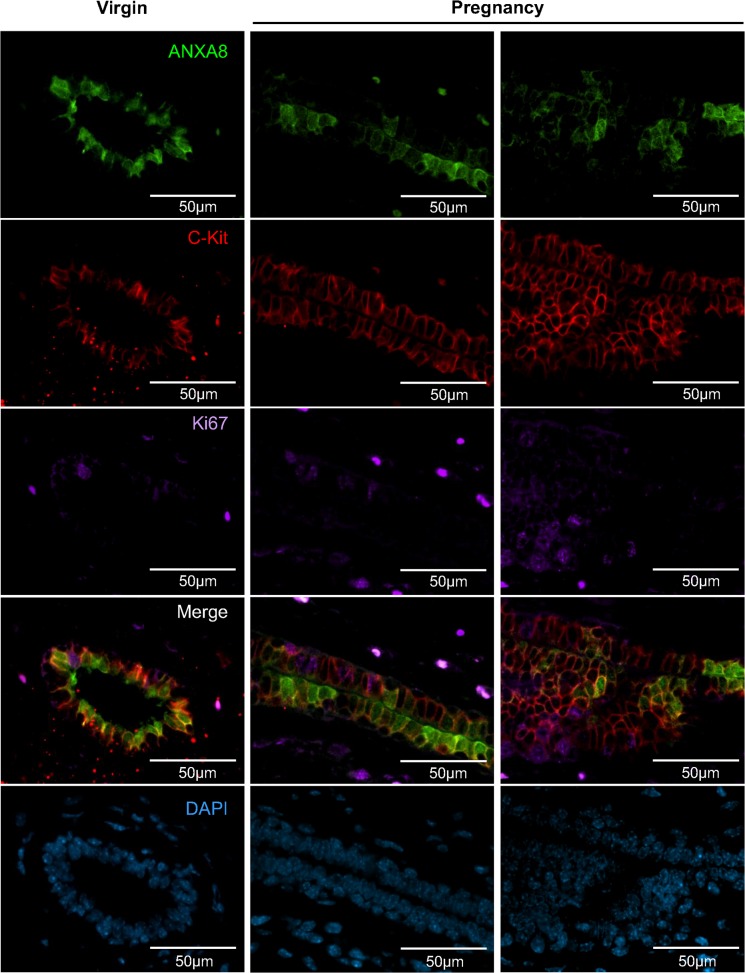
ANXA8+ve/c-kit+ve luminal progenitor cells are mostly Ki67−ve. Co-immunofluorescence staining for ANXA8 (green), c-kit (red), and Ki67 (purple) in mouse mammary gland from virgin and mid-pregnant mice shows that ANXA8+ve cells express c-kit, but not Ki67. The Ki67 staining has been coloured purple for easier visualisation in the triple-merged image. These are representative images of at least three independent mice for each time point.

Triple-staining of mammary glands from pubertal and mid-pregnant mice further confirmed that ANXA8+ve/c-kit+ve cells were mostly Ki67−ve (Fig. 5). Our results therefore strongly suggest that ANXA8 is associated with a subpopulation of mostly quiescent c-kit+ve/ERα−ve ductal luminal progenitor cells.

### ANXA8 over expression reduces proliferation in Kim-2 cells

Since ANXA8 expression was associated with low proliferation in the mammary gland and other tissues [43,44], *in vitro* studies were carried out to assess whether ANXA8 could directly affect cell proliferation. Mouse ANXA8 was over expressed in the mouse mammary epithelial cell line Kim-2, using an inducible episomal vector under the control of doxycycline (dox) (Kim2A8). Approximately 50% of the pooled cells expressed ANXA8 and EGFP through a bidirectional promoter when treated with 100ng/ml dox (S7 Fig.). Since only EGFP-positive cells expressed ANXA8, EGFP-positivity was used as a surrogate marker for ANXA8 expression in further experiments.

**Fig 6:**
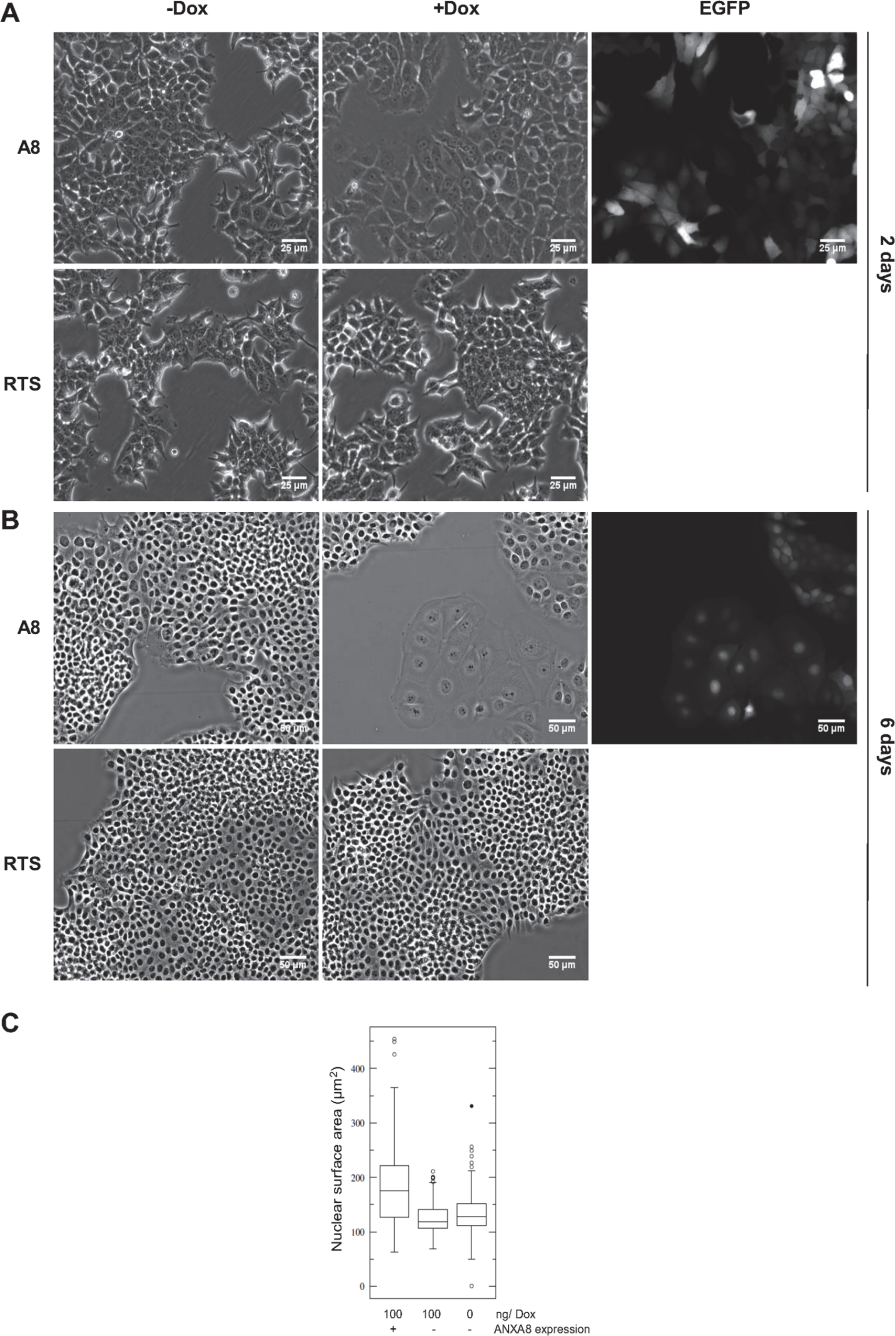
ANXA8 expression induces morphological changes in Kim-2 cells. Kim2A8 and Kim2RTS cells were grown in the presence or absence of 100ng/ml dox. Pictures were taken after 48 hours (**A**) or six days (**B**) of treatment. EGFP was used as a reporter of ANXA8 expression. Both proteins are expressed from opposite sides of a bidirectional promoter. (**C**) Nuclear sizes were analysed after 6 days by measuring the nuclear area (stained with DAPI) of at least 90 individual cells from each dox-treated and untreated populations using ImageJ. There was a significant difference between Kim2A8 cells expressing and not expressing ANXA8 (ANOVA: p<0.05).

Microscopic analysis after dox-treatment showed that after two days the EGFP+ve cells were increased in size (Fig. 6 (A)) compared to EGFP−ve cells, and after six days showed a highly enlarged and flattened morphology (Fig. 6 (B)) with significantly enlarged nuclei (Fig. 6 (C)). Although this morphology was reminiscent of senescent cells, the cells were negative for the senescence markers β-galactosidase (β-gal) and p16 (data not shown).

**Fig 7:**
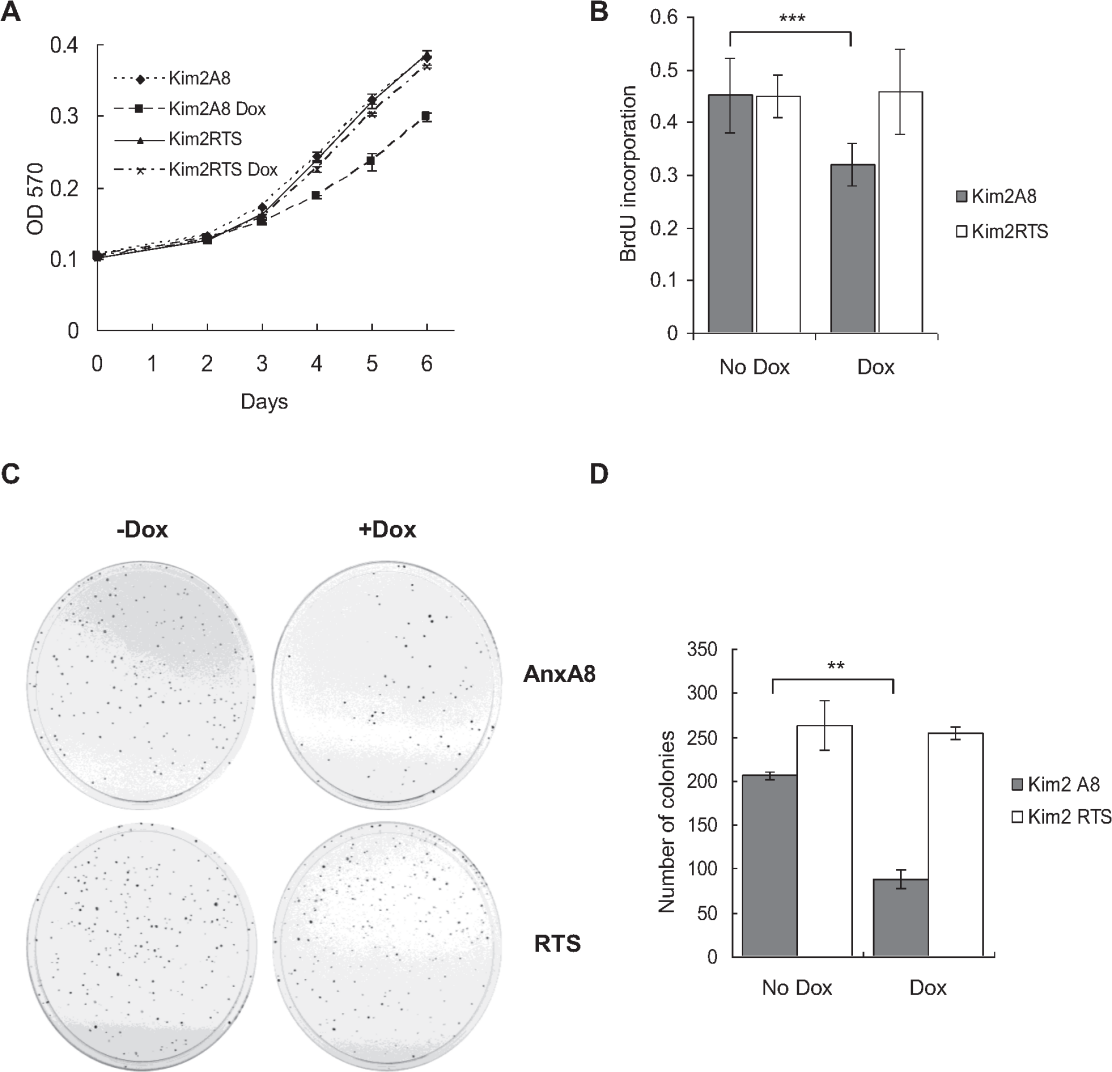
ANXA8 expression inhibits proliferation of Kim2A8 cells. (**A**) Kim2A8 and Kim2RTS cells were seeded in 24-well plates, allowed to attach and grown in the presence or absence of 100ng/ml dox (first treatment at time point 0). At each time point protein extracts were prepared (in duplicates). The graph shows the amount of protein as determined by BCA assay against time. This assay was performed in triplicate and the graph shows a representative result from one experiment. (**B**) Cells were seeded in 96-well plates and treated with dox for 48 hours, labelled with BrdU and the incorporation of BrdU was quantified and plotted for each condition (six wells per condition). ***p< 0.001 (**C**) Equal amount of cells (250–300) were grown in the presence or absence of 100ng/ml dox. After 14 days cells were fixed, stained and the plates photographed. A representative plate per condition is shown. The experiment was carried out in triplicate. (**D**) Graph showing the number of colonies per plate from the experiment (**C**) as quantified by Image J (**p<0.003).

A cell growth assay of Kim2A8 cells showed that after three days of dox-treatment Kim2A8 cell growth was significantly reduced compared to control cells (Fig. 7 (A)). Since the decreased growth rate was associated with reduced BrdU incorporation (Fig. 7 (B)) our results showed that in this system ANXA8 over-expression was able to reduce cell proliferation.

### ANXA8 over expression prevents colony formation of Kim-2 cells *in vitro*

To further characterise the effect of ANXA8 over expression on cell proliferation, a 2D colony formation assay was performed. Kim2A8 and control cells were seeded as single cell suspensions and treated with or without dox. Colonies were analysed by bright-field and fluorescence microscopy. After two weeks of dox-treatment Kim2A8 cells formed significantly fewer colonies of more than 50 cells compared to untreated Kim2A8 cells or control cells (Fig. 7 (C, D)). All large colonies that formed from the dox-treated Kim2A8 cells were largely EGFP−ve (with the occasional entrapped green cell), and hence did not express ANXA8. EGFP+ve cells remained either as single cells or very small colonies of <10 cells, and showed again the above mentioned enlarged morphology (S8 Fig.).

### ANXA8 over expression induces quiescence in Kim-2 cells

**Fig 8:**
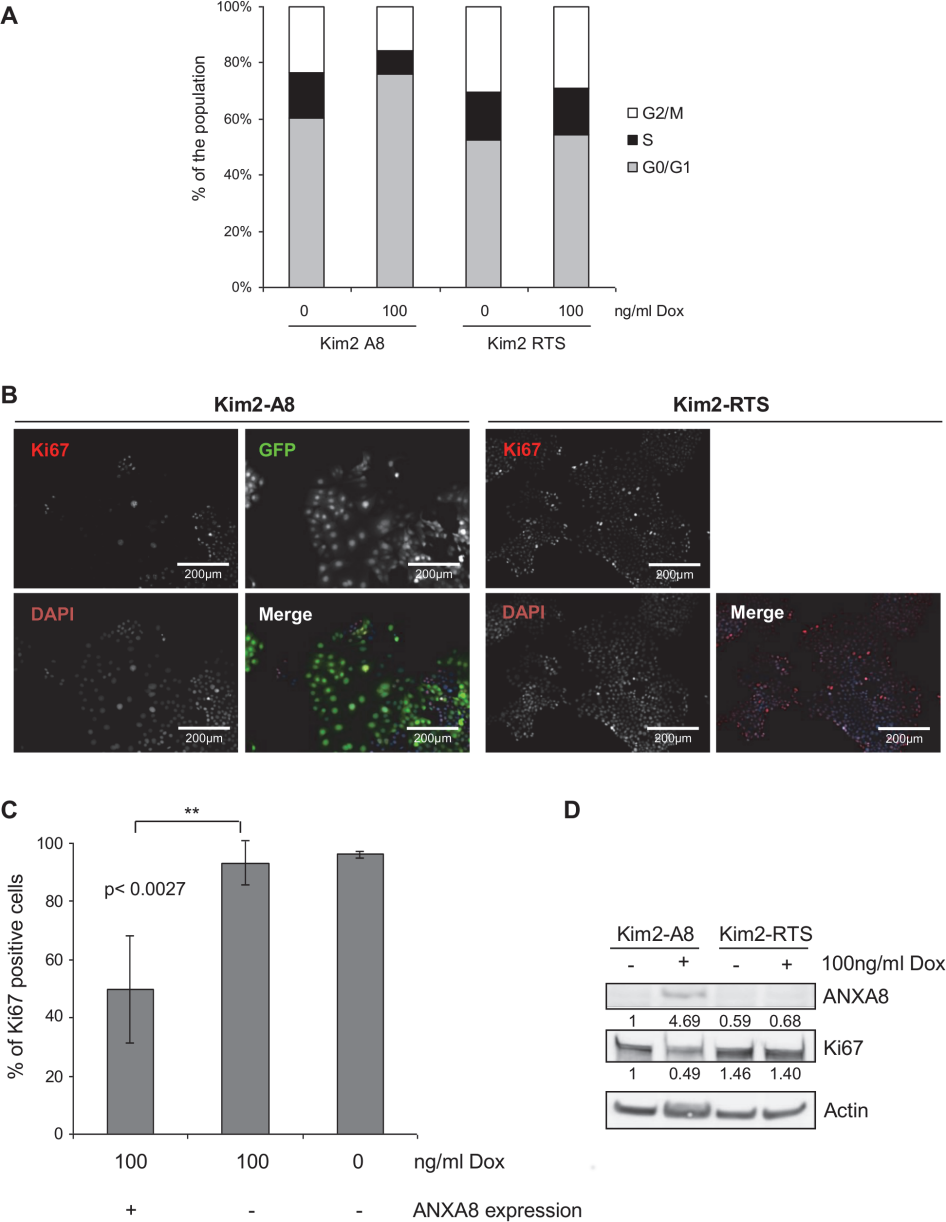
ANXA8 expression arrests Kim2A8 cells in G_0_. (**A**) Kim2A8 and Kim2RTS cells were grown in 6-well plates with or without 100ng/ml of dox for 48 hours. The graph shows the average percentage numbers of cells in G_0_/G_1_, S and G_2_/M as quantified by FACS from three independent experiments. (**B**) Kim2A8 and Kim2RTS cells grown in chamber slides with or without 100ng/ml dox for six days were fixed and stained for Ki67 antigen. EGFP was used as a reporter of ANXA8 expression. (**C**) Graph showing the percentage of Ki67-positivity in the EGFP positive and negative populations of Kim2A8 cells grown with or without 100ng/ml dox. At least 1000 cells were analyzed in each population. (**D**) Western blot showing Ki67 and ANXA8 protein expression in cells after six days in culture. Actin was used as a loading control. Numbers show the relative intensities of ANXA8 and Ki67 bands respectively (normalised to actin) determined by measuring area pixel intensities using AIDA Image Analyzer software. The reduction of Ki67 levels (∼50%) is consistent with the reduced number of Ki67+ve Dox-treated Kim2A8 cells seen in (**B**).

FACS analysis was performed after 48 hours with or without dox-treatment to further assess the nature of the growth arrest induced by ANXA8 expression in Kim2A8 cells (Fig. 8 (A)). Dox-treated Kim2A8 cells showed a significantly higher proportion of cells in G_0_/G_1_ (75%) compared to untreated Kim2A8 cells (59%) or the negative control cells (52% −dox; 54%+dox), while the amount of Kim2A8 cells in S- or G_2_/M-phase was strongly reduced (S-phase: −dox: 16%; +dox:<8%; G_2_/M: −dox:23% vs +dox:15%), demonstrating an arrest at G_0_/G_1_. No increase in the sub-G_0_/G_1_ fraction was observed (all between 1–1.5%), confirming that ANXA8 over expression did not induce cell death.

To establish whether ANXA8 over expressing cells were arrested in G_1_ or entered G_0_, we measured Ki67 expression levels by IF staining (Fig. 8 (B, C)) and western blot (Fig. 8 (D)). IF staining showed that only ∼50% of EGFP-positive Kim2A8 cells expressed Ki67, while nearly all EGFP negative cells, like the control cells, were Ki67 positive (∼95%; p<0.003). In western blots untreated cells and dox-treated control cells showed similar levels of Ki67 protein, while over expression of ANXA8 for 6 days significantly reduced Ki67 protein levels by ∼50%. This demonstrated that ANXA8 over expression had taken KIM-2 cells out of the cell cycle.

## Discussion

We previously described Anxa8 mRNA to be expressed in isolated mammary ducts and after enforced involution, but not during pregnancy and lactation when primary ducts branch and bud to form milk secreting alveoli [45]. The current study further defines ANXA8 expression in a mostly quiescent subpopulation of c-kit+ve/ERα−ve progenitor cells of the ductal epithelium, and identifies ANXA8 as a potential regulator of proliferation and/or quiescence in the mouse mammary ductal epithelium. No significant levels of AnxA8 expression were found in ERα+ve cells, or in differentiated alveoli of late pregnant or lactating mice (Figs. 1, and 3), showing that ANXA8 expression was not associated with terminal differentiation of the mammary epithelium. Instead, ANXA8 was only present in the major ducts, which bud during pregnancy to form alveoli, and was widespread in the ducts of a late involuting gland, when apoptosis had ceased and the primary mammary ductal system was regenerating.

Although a large proportion of c-kit+ve cells showed ANXA8 expression by double-IF staining, not all of them did, and as ANXA8 and Ki67 staining were largely exclusive it appears that ANXA8 may be characteristic of a mostly quiescent subpopulation of committed ERα−ve progenitor cells, which our triple-staining supports. However, rare Ki67+ve/ANXA8+ve cells could be detected (less than 1% of all ANXA8+ve cells) during puberty and this proportion increased at the start of pregnancy, but decreased again with prolonged pregnancy while Ki67 staining of ductal ANXA8−ve cells remained increased. Therefore, although ANXA8 expressing cells were mostly quiescent, these cells were not terminally differentiated. It is unclear whether the progeny of the ANXA8+ve cells contribute to the formation of side branches and alveoli. No ANXA8 staining was found in alveoli and c-kit staining was equally down-regulated. Though *AnxA8* mRNA was recently detected in quiescent normal human mammary stem cells isolated from mammosphere cultures on the basis of PKH26-retention [46], we could not detect ANXA8 in mouse mammary stem cells. An association with very early mammary epithelial progenitor cells is supported by the recent finding that *AnxA8* mRNA is expressed in the early developing mammary bud epithelium (E12.5) which showed limited proliferation and expressed c-kit [47], though in contrast to c-kit we were not able to detect ANXA8 protein by IHC at this time point (data not shown).

A recent finding that ANXA8 is part of the ADAM-17/AREG shedding complex and can modulate the shedding of pro-amphiregulin and other EGF family members on the cell surface [48], and the finding that ANXA8 affects ligand-induced degradation of EGFR [31] raises the attractive possibility that ANXA8 may directly affect ligand availability for growth factor receptors and/or signalling, thereby controlling cell growth and/or differentiation. Since ADAM17 has also been shown to be a major sheddase for c-kit [49] and growth factor ligands including kit-ligand [50] it is tempting to speculate that ANXA8 may directly affect c-kit signalling in the luminal progenitors of the mammary gland. It was also noticeable that the number of ANXA8 positive cells varied greatly between ductal areas in a gland and between glands of similar time points. Whether ANXA8+ve cells mark the sites of future secondary/tertiary branches is currently subject of further investigations using a lineage tracing approach.

Our current data are further consistent with other studies that have linked *AnxA8* expression to reduced proliferative activity and/or quiescence. When quiescent NIH3T3 fibroblasts were driven into proliferation by transduction with an adenoviral E2F1 construct *Anxa8* was one of the strongest down-regulated genes in two independent experiments [51]. Similar results for *Anxa8* were obtained when NIH3T3 cells were transfected with Nanog, leading to increased proliferation and transformation and a reduction in *AnxA8* mRNA [52]. In the fetal bovine growth plate ANXA8 expression forms a gradient in which the higher expression is found in the low-proliferative hypertrophic zone [44], while in adult mouse stratified epithelia ANXA8 is expressed in supra-basal layers, suggesting that ANXA8 expression may be associated with partial differentiation [43], though our own studies identified ANXA8 in Ki67−ve cells of the basal layer (data not shown). Further, since ANXA8 expression was not detected in differentiating alveolar cells during pregnancy or lactation it is highly unlikely to be associated with differentiation in the mammary gland.

Despite *AnxA8* mRNA up-regulation early during involution, ANXA8 protein could not be detected in the early collapsing alveoli and therefore was unlikely to be involved in apoptosis, as our microarray profile may have suggested. Involution can be induced through forced weaning of the pups at the height of lactation, leading to widespread alveolar cell death and tissue remodeling, after which the mammary gland resembles a pre-pregnant-like mammary gland. Transcriptional microarray profiling identified *Anxa8* mRNA to be strongly increased 24 hours after enforced mammary gland involution with sustained abundance for several days [29], though our qRT-PCR data now show a much slower increase. Similar increases can be found for c-kit and the kit ligand SCF (S9 Fig.), indicating that the involution recovery involves c-kit+ve progenitor cells and/or leads to a relative increase in c-kit+ve cells due to a preferential loss of differentiated alveolar cells. ANXA8 protein was not detected in the apoptotic alveoli 48 hours after enforced weaning (Fig. 1, S1 Fig.), but was detectable in major ducts and in most of the surviving epithelium after 10 days. Neither could ANXA8 be found in TEB during puberty where apoptosis is prevalent during ductal lumen formation [53,54]. Our view that ANXA8 is not associated with cell death is further supported by our finding that ANXA8 over expression in Kim-2 cells did not induce cell death, as indicated by no change in the sub-G_1_ fraction after ANXA8 over expression (Fig. 8). There was though some expression of ANXA8 in the collapsed epithelial structures 4 days after forced weaning when most apoptosis has already ceased and tissue remodeling with an immune response and suppressed inflammation occurs [35,55,56]. It cannot be ruled out that ANXA8 expression in these structures may be associated with its PLA_2_ inhibitory activity described for many annexins, including ANXA8 [17], thereby supporting the inflammatory suppression described previously [35,56].

Several annexins, including annexins A1 [10,57], A2 [58,59] and A6 [60,61] have been found to modulate proliferation or to be directly involved in cell division, including annexin A11, which is part of and necessary for midbody formation during cytokinesis [5]. Inhibition of proliferation induced by ANXA1 and ANXA6 correlated with concomitant changes in the actin cytoskeleton and cell morphology. ANXA8 has previously been found to interact with F-actin in co-sedimentation assays and with PIP_2_, suggesting that ANXA8 may play a role in the regulation of actin/membrane interactions [62]. However, we did not detect any changes in the actin cytoskeletal structure in Kim2A8 cells (data not shown). The morphological changes we observed were similar to those reported for ANXA2 over-expression in MIO Müller cells [63], in which ANXA2 expression induced the cells to become flattened, but the increase in the size of the footprint observed did not correlate with an increase in cellular volume when analysed by FACS. It has also been demonstrated that over expression of ANXA8 in SCK, MDA-MB231 and NIH3T3 cells can induce morphological changes, inducing a more epithelial-like morphology, and that these changes may be mediated through direct interaction of ANXA8 with FAK [23].

ANXA8’s reduced expression in the majority of breast cancers is consistent with an anti-proliferative role of ANXA8. However, the finding that positive staining correlates with basal-like breast cancers that are of high grade and positive for Ki67 [29] is somewhat counterintuitive. Similar results have been described for the tumour suppressor protein p16/INK4, which is associated with cell cycle arrest and senescence, but is strongly associated with basal-like breast cancers [64]. Further, the cell cycle protein cyclin E has been shown to induce cell cycle arrest by p27KIP accumulation in mammary epithelial cell lines HC-11 and 184B5, though it increased proliferation in others [65,66] and is strongly expressed in basal-like breast cancers [67]. Since c-kit+ve/ERα−ve luminal progenitor cells have recently been shown to be the origin of basal-like breast cancers [42,68], and since ANXA8 is strongly associated with this subgroup, it is possible that ANXA8 expression in these cancers is a reflection of ANXA8’s close association with luminal progenitor cells, and that the pro-quiescence function of ANXA8 might be perturbed. A similar association of ANXA8 with committed human progenitor cells and cancer had previously been found in the haematopoietic system, where *AnxA8* mRNA expression was detected in pro-myelocytes and was further up-regulated in APL [69], a leukaemia in which the c-kit+ve progenitor cell population is abnormal and expanded. AnxA8 was specifically up-regulated in APL but not in other myelocytic leukaemias [18,19,69]. Treatment with ATRA down-regulated ANXA8 expression and pushed cells into differentiation [19]. Though a causal link between ANXA8 down-regulation and differentiation of APL cells has not been tested it is tempting to speculate that ANXA8 might be involved in progenitor cell maintenance. Further studies will reveal whether the same association exists between the ANXA8+ve cells of the terminal duct lobular unit [29] and c-kit in the human breast.

Given our results and the association of ANXA8 expression with ER−ve breast cancers it will be worth testing whether ANXA8 over-expression in ER+ve breast cancer cell lines can induce cellular quiescence or reduce proliferation. However, as the human genome contains at least two *Anxa8* genes (*Anxa8*, *Anxa8l1*), which vary slightly in their encoding sequence, and in the absence of promoter expression studies, epigenetic analyses and human genotyping, it is impossible to know which one (if not both) of these genes contributes to the variations observed in gene expression in the normal and malignant breast. The existence of copy number and allelic population variants could further affect gene product "dosage" as well as the pharmacogenetic responsiveness of these genes [70]. Therefore, over-expression of each variant on its own and in combination in a variety of breast cancer cell lines will be necessary.

## Conclusion

We have established for the first time that ANXA8 expression is associated with a subpopulation of transiently quiescent c-kit+ve/ERα−ve cells of the ductal epithelium and that ANXA8 over expression can induce quiescence *in vitro*. The mechanism(s) by which ANXA8 induces this G_0_-arrest is still unknown. Its expression is therefore strongly associated with a luminal epithelial progenitor cell population that is thought to be the origin of basal-like breast cancers, a subgroup of breast cancers with which ANXA8 is strongly associated. Further work will establish whether ANXA8 is functionally involved in progenitor cell quiescence and/or maintenance, and whether ANXA8 positive mammary epithelial cells may be the origin of ANXA8-expressing basal-like breast cancers.

## Supporting Information

S1 Fig ANXA8 protein expression during mammary gland development. Close-up view of images from Fig. 1. The black bar represents 100μm.
(TIF)

S2 Fig qRT-PCR results for AnxA8 from 13 stages of mammary gland development. Total RNA was extracted from mammary glands at the indicated time points and tested for presence of Anxa8 mRNA by qRT-PCR. Results are shown in relation to Krt 18 mRNA abundance.
(TIF)

S3 Fig ANXA8 and Ki67 expression in TEB, ducts, and during early pregnancy. Immunohistochemical staining for ANXA8 and Ki67 of consecutive mammary tissue sections of the same TEB and duct region from the same 3-, and 5-week C57BL/6 old pubertal mice, and from an early pregnant mouse (day 4.5) shows that areas of ANXA8 and Ki67 expression are largely exclusive. Magnification x100.
(TIF)

S4 Fig ANXA8 expression in EdU labelled mammary glands. Double-immunofluorescent labelling for ANXA8 (green) and EdU (red) shows that ANXA8+ve cells are EdU−ve in pre-puberty and during involution. Mouse mammary glands have been *in vivo* labelled for 2 hours in pre-pubertal mice (3 weeks of age) (**A**), and 4 days after forced weaning (**B**) before culling. (**A**) Top two rows show examples of mammary ducts with high ANXA8-staining but little EdU staining in the mammary epithelium, while the bottom row shows a typical TEB with high EdU-staining but no ANXA8 staining. (**B**) At 4 days of involution mammary glands showed no epithelial EdU incorporation, but widespread ANXA8 expression. Top two rows show two epithelial ducts, while the bottom row shows positive EdU staining in lymphocytes of the inguinal lymph node (pos. control). Bars represent 50μm.
(TIF)

S5 Fig ANXA8 positive cells are negative for MCM3. Co-immunofluorescence staining for ANXA8 and MCM3 in 6-week old C57BL/6 mice shows that those cells strongly positive for ANXA8 are MCM3−ve. Bars represent 50μm.
(TIF)

S6 Fig Co-expression of ANXA8 and c-kit protein. Co-immunofluorescence staining for ANXA8 (red), and c-kit (green) in a mouse mammary gland from a 6-week old virgin (V6) and a 12-day pregnant (P12.5) adult mouse showing that while all ANXA8+ve cells express c-kit, only a subgroup of c-kit+ve cells express ANXA8. Bars represent 50μm.
(TIF)

S7 Fig Kim2A8 cells express ANXA8 and EGFP after dox induction. (**A**) Kim2A8 cells were grown in chamber slides with 100ng/ml dox for 24 hours, fixed and stained with E2R6.2 antibody to detect ANXA8 expression. EGFP was co-expressed by a bi-directional promoter. All EGFP positive cells expressed ANXA8, so that EGFP positivity could be used as a reporter for ANXA8 expression in this cell line. (**B**) Kim2A8 and Kim2RTS cells were grown in the presence of 100ng/ml of dox for 5 days and ANXA8 protein levels measured in dox-treated and un-treated cells. Actin was used as a loading control.
(TIF)

S8 Fig Colony formation of ANXA8 over-expressing Kim2 cells is suppressed. Kim2A8 cells were grown for two weeks in the presence of 100ng/ml dox as described in Fig. 7(C). Single cells or small colonies (<20 cells) of EGFP-positive Kim2A8 cells were detected after two weeks of growth. These cells showed a flat, large and round morphology. Images of typical colonies from Kim2A8 cells with or without dox treatment are shown.
(TIF)

S9 Fig RNA expression of *c-kit*, *SCF* and *AnxA8* during enforced involution. Microarray results from lactating (day 7) and involuting (days 1, 2, 3, 4, 20) mouse mammary glands from a previous study [35]. The graphs show the normalized average signal intensities for *AnxA8*, *c-kit*, and *scf/kit ligand* mRNAs ±standard error.
(TIF)
