# Supplementary figures and images for: Annexin A8 Identifies a Subpopulation of Transiently Quiescent c-Kit Positive Luminal Progenitor Cells of the Ductal Mammary Epithelium
Source: PLoS One. 2015 Mar 24;10(3):e0119718. doi: 10.1371/journal.pone.0119718 (PMC4372349; doi:10.1371/journal.pone.0119718)

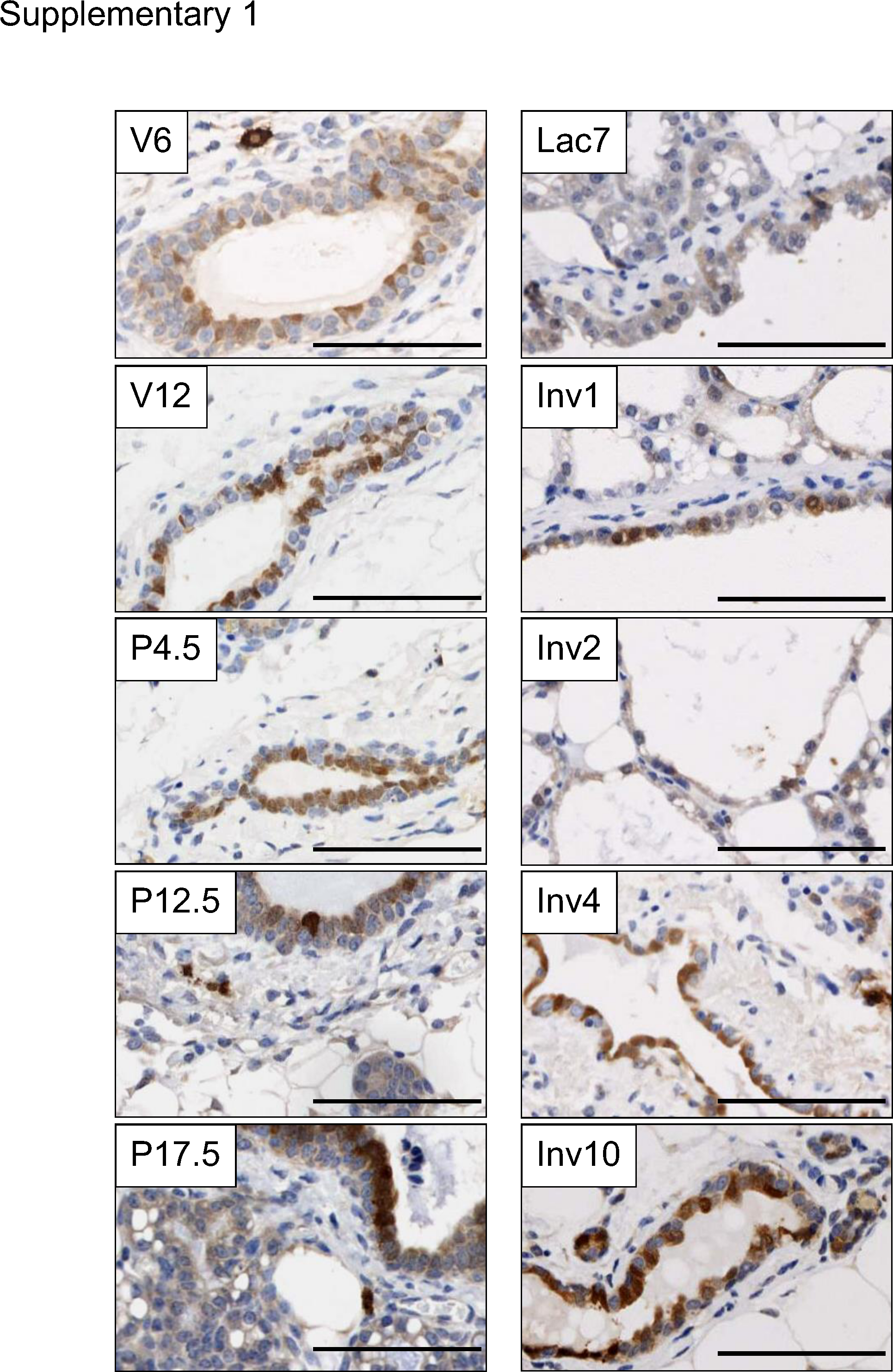

Supplement: S1 Fig — Close-up view of images from Fig. 1. The black bar represents 100μm. (TIF) [file pone.0119718.s001.tif]

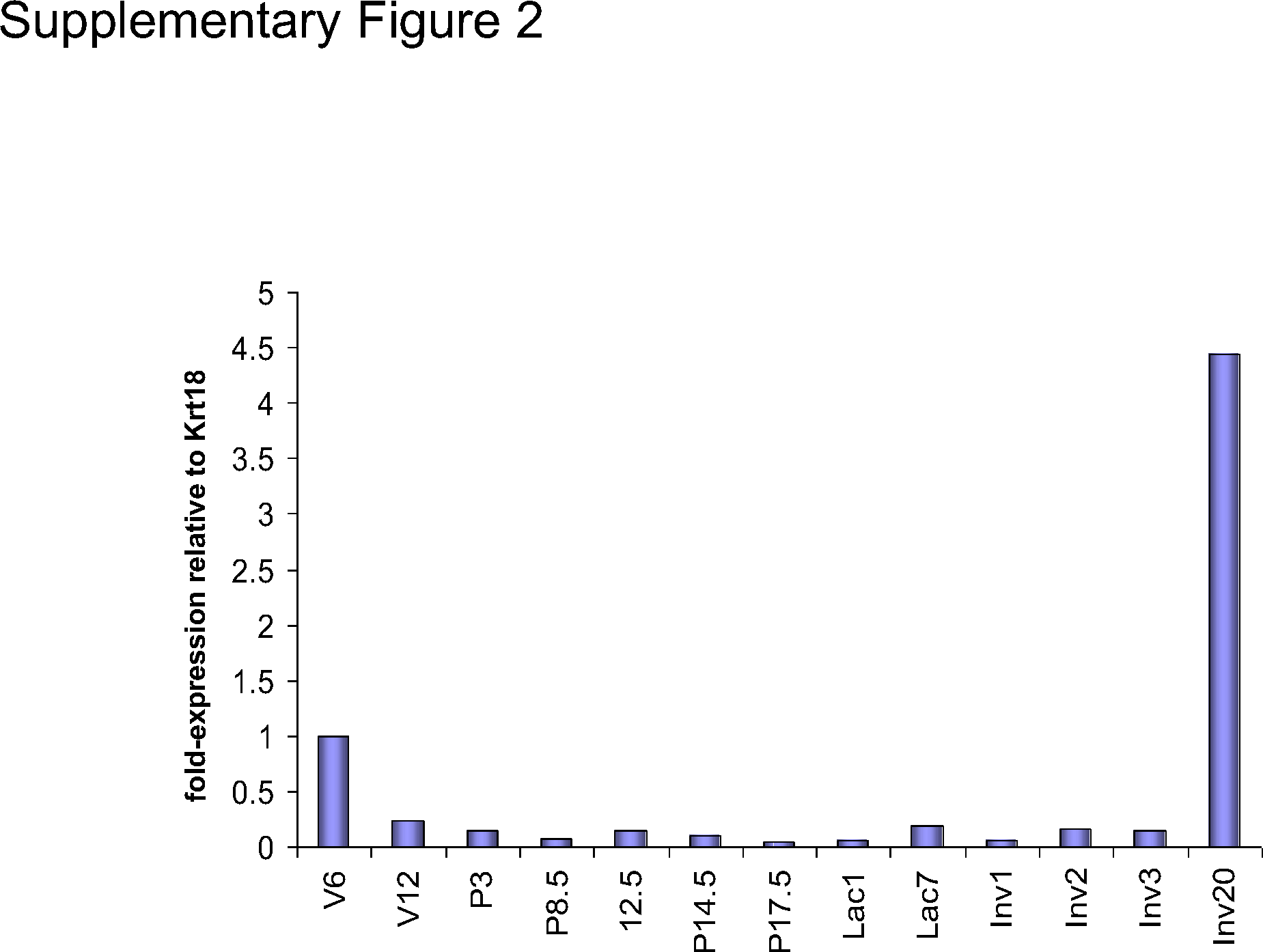

Supplement: S2 Fig — Total RNA was extracted from mammary glands at the indicated time points and tested for presence of Anxa8 mRNA by qRT-PCR. Results are shown in relation to Krt 18 mRNA abundance. (TIF) [file pone.0119718.s002.tif]

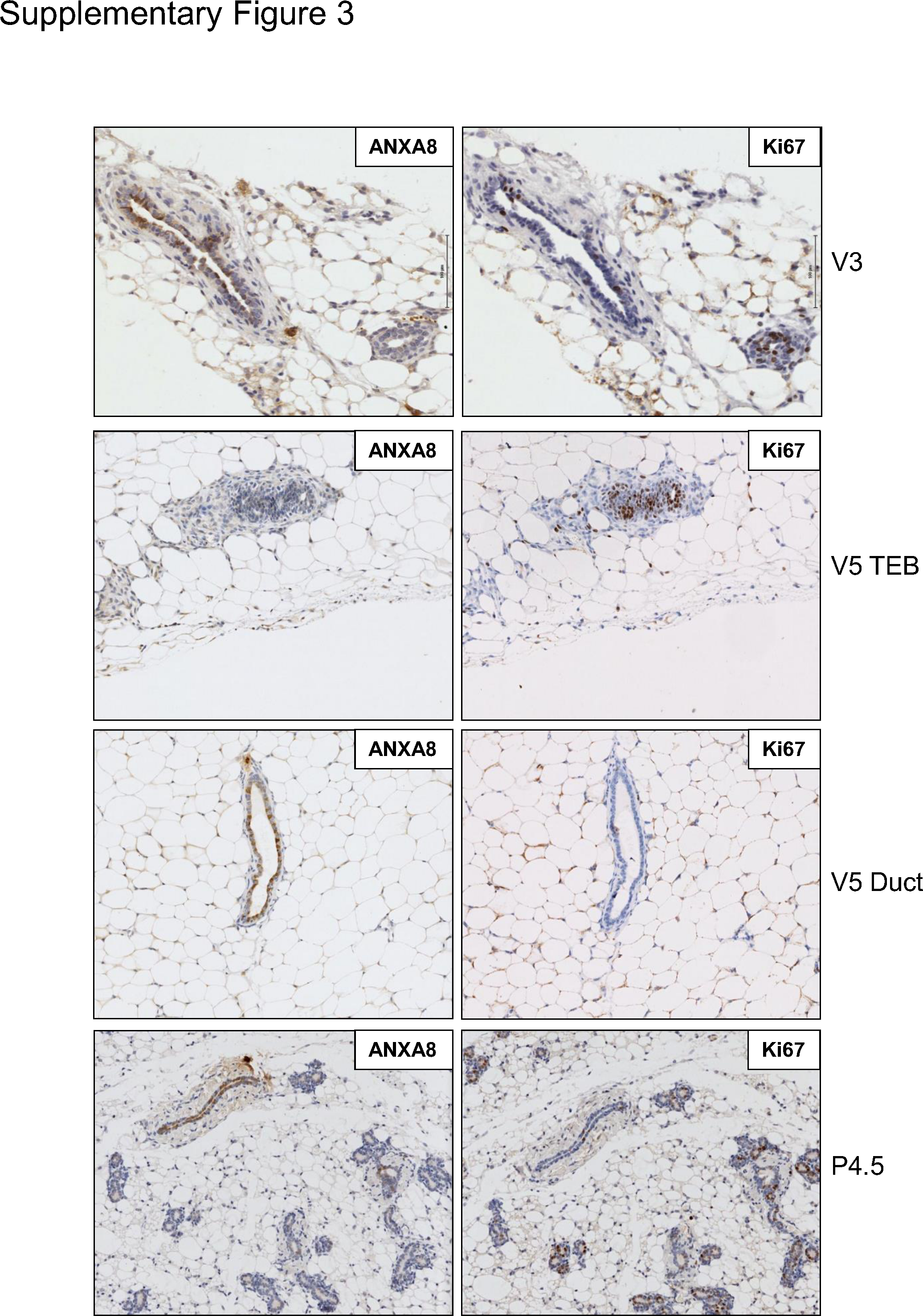

Supplement: S3 Fig — Immunohistochemical staining for ANXA8 and Ki67 of consecutive mammary tissue sections of the same TEB and duct region from the same 3-, and 5-week C57BL/6 old pubertal mice, and from an early pregnant mouse (day 4.5) shows that areas of ANXA8 and Ki67 expression are largely exclusive. Magnification x100. (TIF) [file pone.0119718.s003.tif]

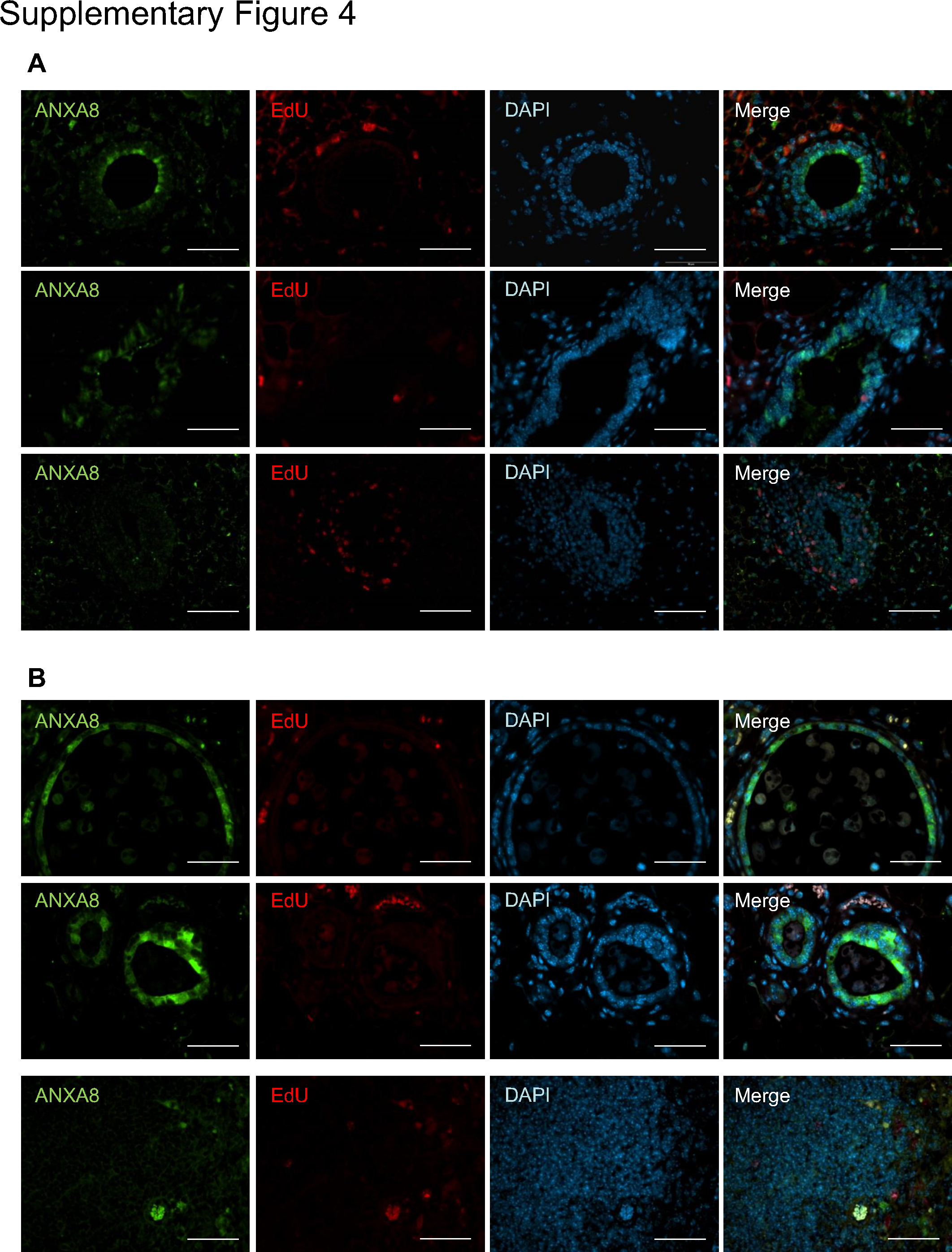

Supplement: S4 Fig — Double-immunofluorescent labelling for ANXA8 (green) and EdU (red) shows that ANXA8+ve cells are EdU−ve in pre-puberty and during involution. Mouse mammary glands have been in vivo labelled for 2 hours in pre-pubertal mice (3 weeks of age) (A), and 4 days after forced weaning (B) before culling. (A) Top two rows show examples of mammary ducts with high ANXA8-staining but little EdU staining in the mammary epithelium, while the bottom row shows a typical TEB with high EdU-staining but no ANXA8 staining. (B) At 4 days of involution mammary glands showed no epithelial EdU incorporation, but widespread ANXA8 expression. Top two rows show two epithelial ducts, while the bottom row shows positive EdU staining in lymphocytes of the inguinal lymph node (pos. control). Bars represent 50μm. (TIF) [file pone.0119718.s004.tif]

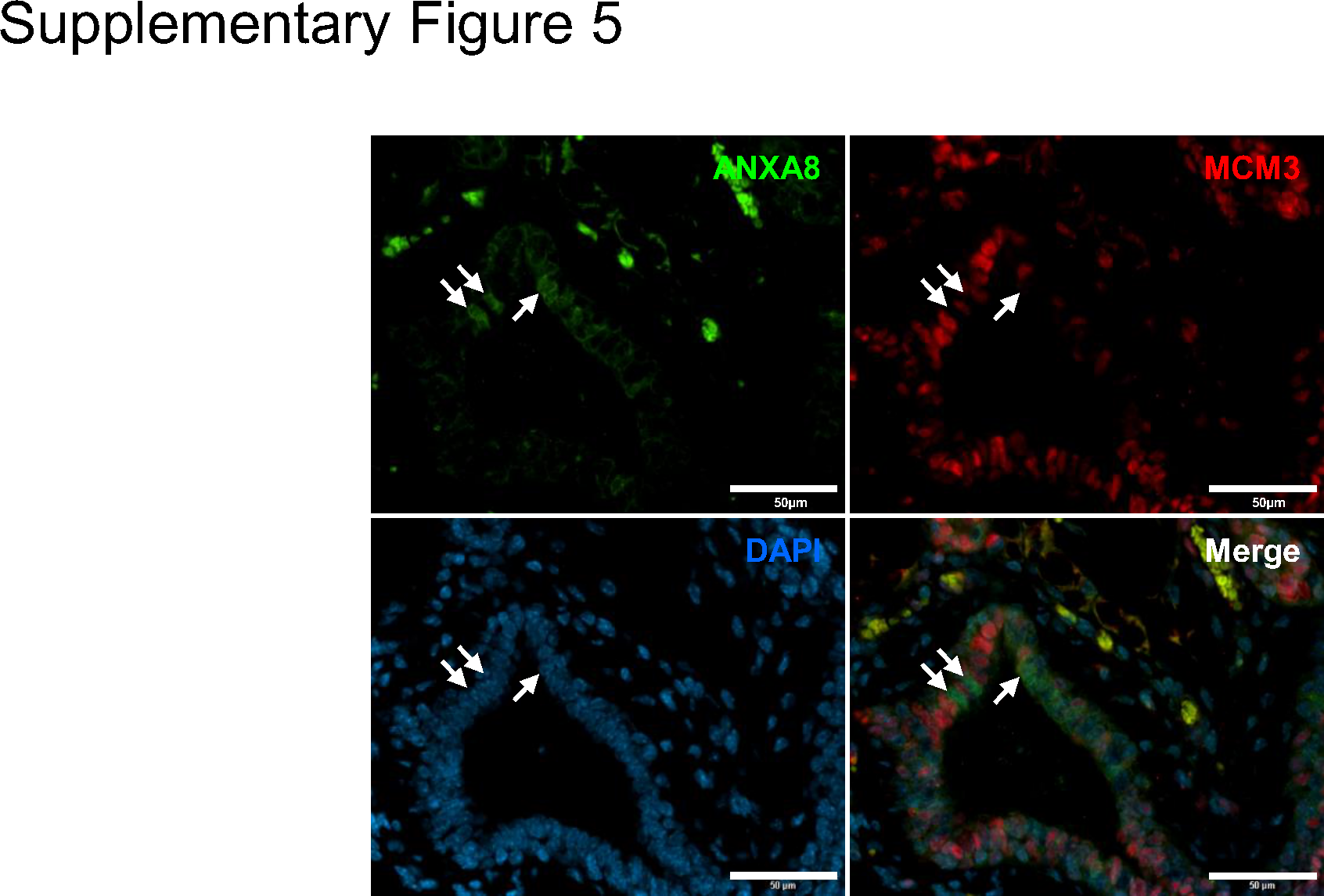

Supplement: S5 Fig — Co-immunofluorescence staining for ANXA8 and MCM3 in 6-week old C57BL/6 mice shows that those cells strongly positive for ANXA8 are MCM3−ve. Bars represent 50μm. (TIF) [file pone.0119718.s005.tif]

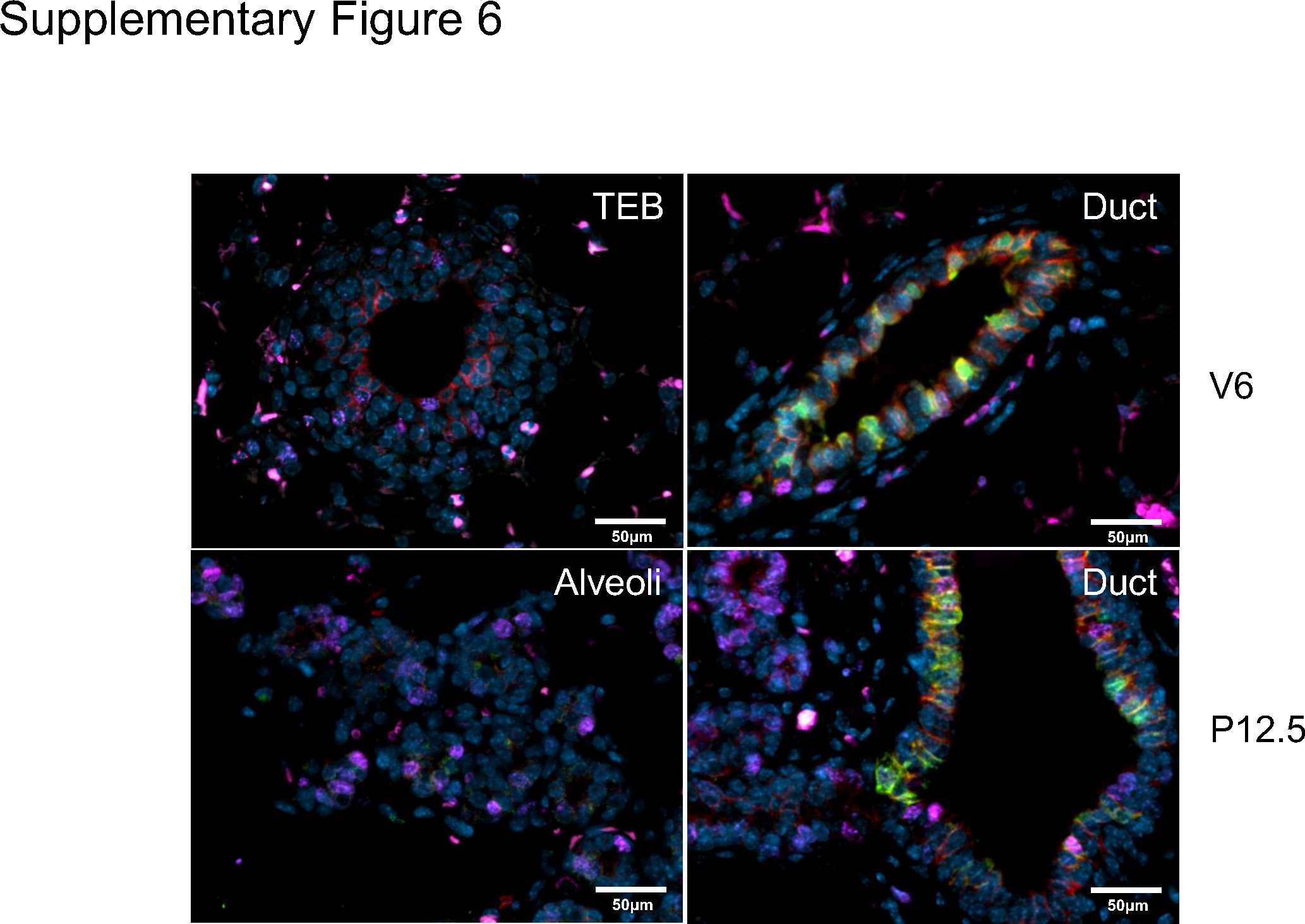

Supplement: S6 Fig — Co-immunofluorescence staining for ANXA8 (red), and c-kit (green) in a mouse mammary gland from a 6-week old virgin (V6) and a 12-day pregnant (P12.5) adult mouse showing that while all ANXA8+ve cells express c-kit, only a subgroup of c-kit+ve cells express ANXA8. Bars represent 50μm. (TIF) [file pone.0119718.s006.tif]

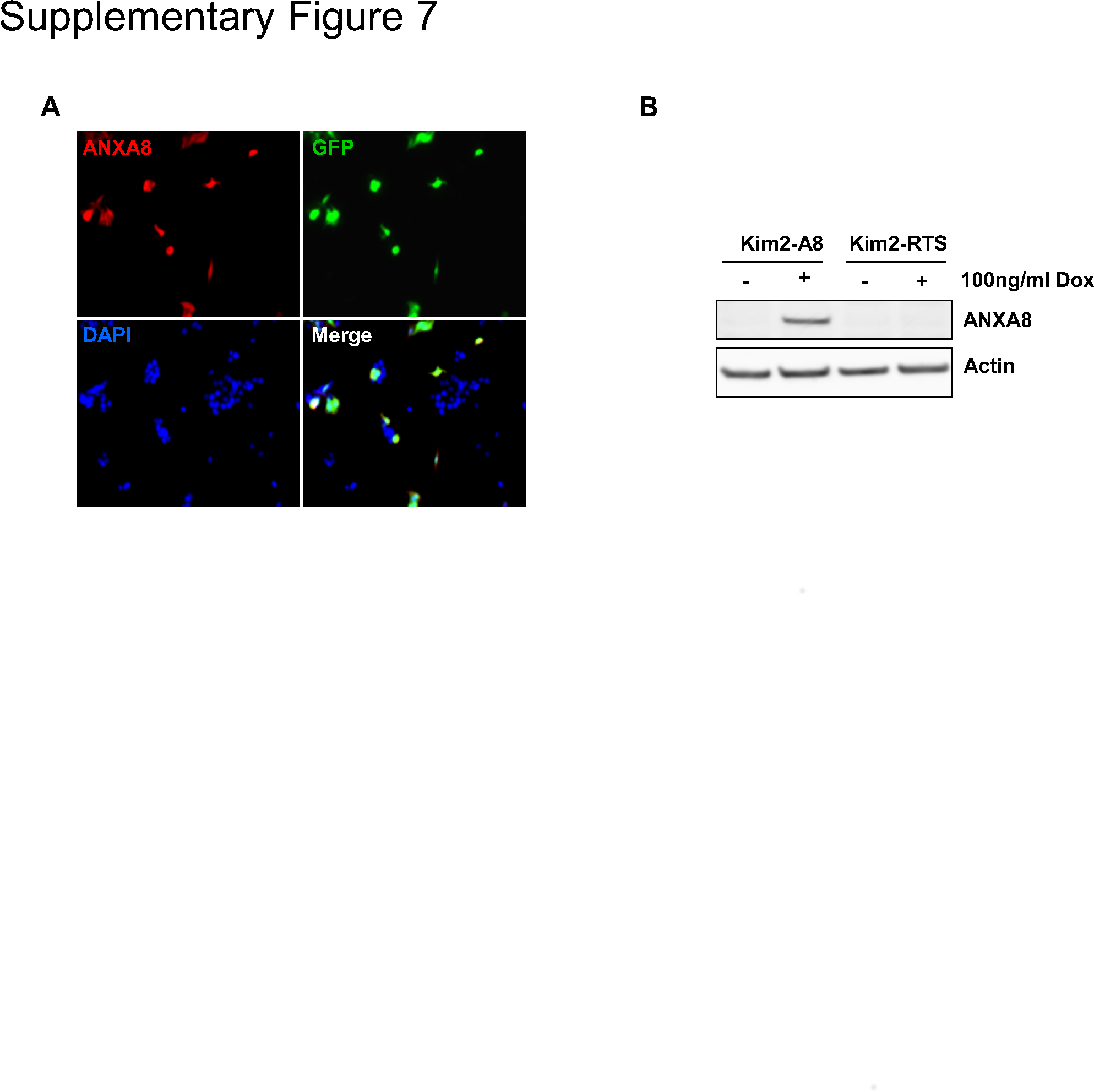

Supplement: S7 Fig — (A) Kim2A8 cells were grown in chamber slides with 100ng/ml dox for 24 hours, fixed and stained with E2R6.2 antibody to detect ANXA8 expression. EGFP was co-expressed by a bi-directional promoter. All EGFP positive cells expressed ANXA8, so that EGFP positivity could be used as a reporter for ANXA8 expression in this cell line. (B) Kim2A8 and Kim2RTS cells were grown in the presence of 100ng/ml of dox for 5 days and ANXA8 protein levels measured in dox-treated and un-treated cells. Actin was used as a loading control. (TIF) [file pone.0119718.s007.tif]

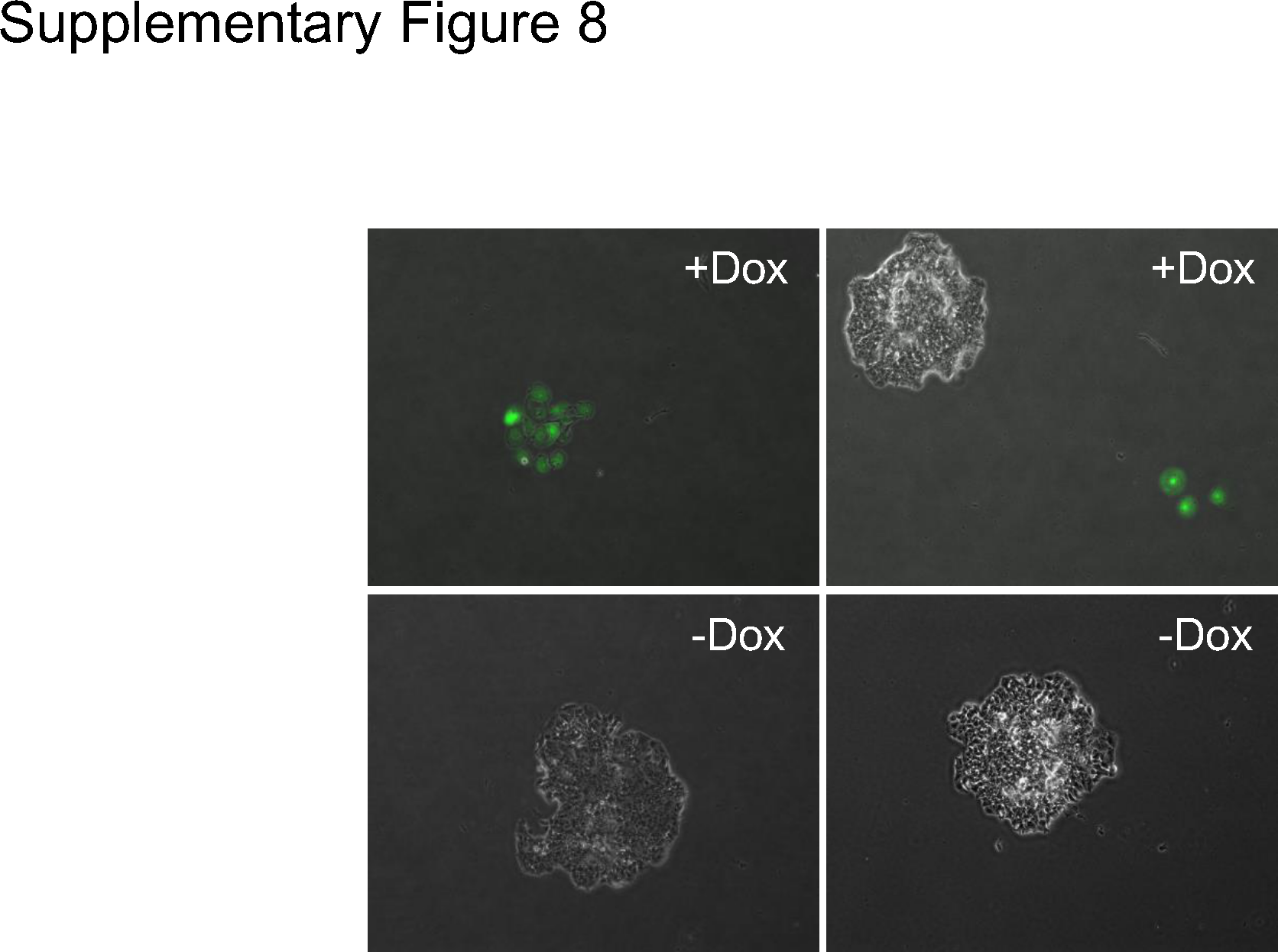

Supplement: S8 Fig — Kim2A8 cells were grown for two weeks in the presence of 100ng/ml dox as described in Fig. 7(C). Single cells or small colonies (<20 cells) of EGFP-positive Kim2A8 cells were detected after two weeks of growth. These cells showed a flat, large and round morphology. Images of typical colonies from Kim2A8 cells with or without dox treatment are shown. (TIF) [file pone.0119718.s008.tif]

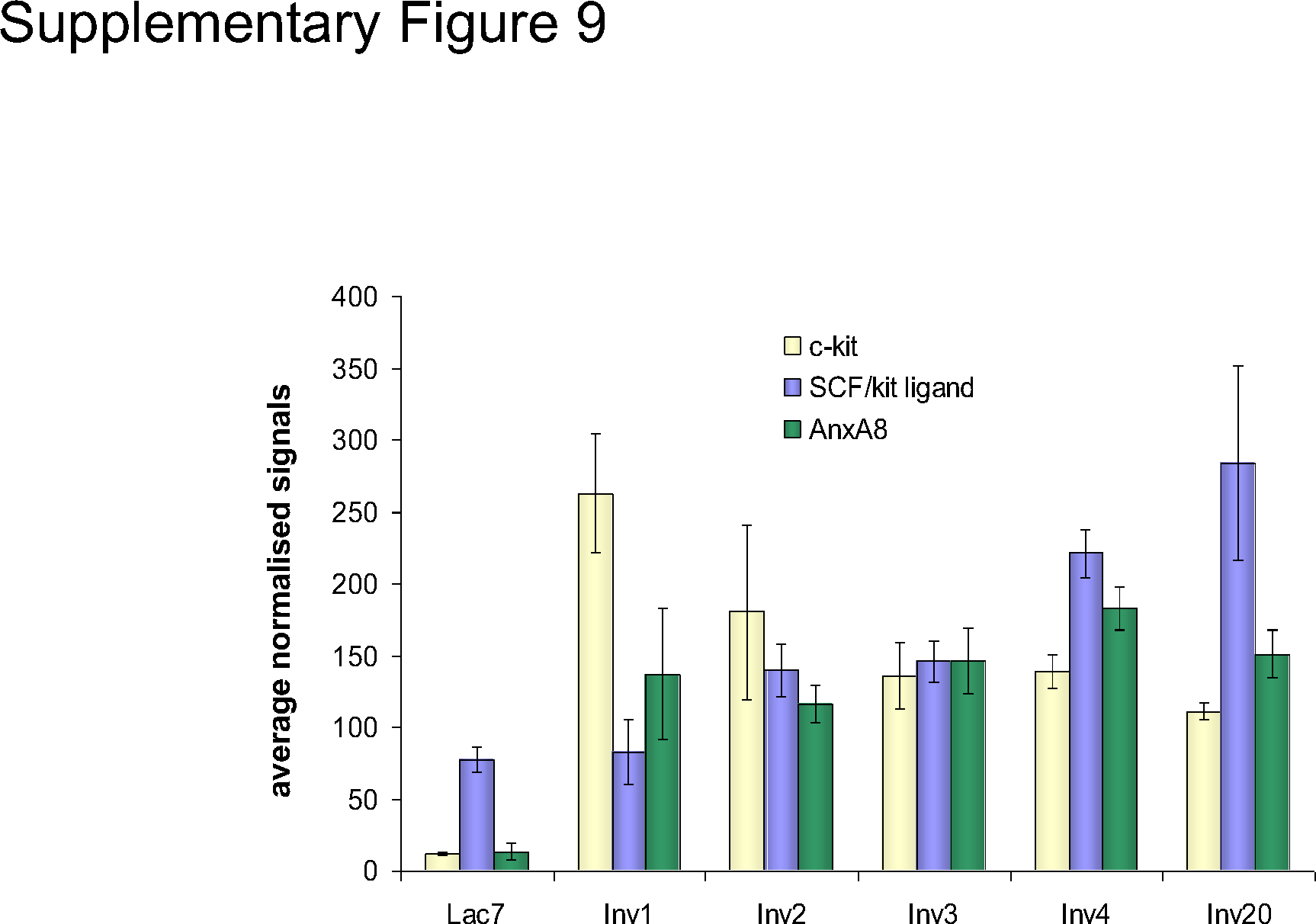

Supplement: S9 Fig — Microarray results from lactating (day 7) and involuting (days 1, 2, 3, 4, 20) mouse mammary glands from a previous study [35]. The graphs show the normalized average signal intensities for AnxA8, c-kit, and scf/kit ligand mRNAs ±standard error. (TIF) [file pone.0119718.s009.tif]
